# Single-Molecule Junction Formation in Deep Eutectic Solvents with Highly Effective Gate Coupling

**DOI:** 10.1021/acs.jpcc.3c03129

**Published:** 2023-06-27

**Authors:** Xiaohang Qiao, Andrea Vezzoli, Shaun Smith, Simon J. Higgins, Ross J. Davidson, Andrew Beeby, Richard J. Nichols

**Affiliations:** † Department of Chemistry, University of Liverpool, Crown St, Liverpool L69 7ZD, U.K.; ‡ Department of Chemistry, Durham University, South Rd, Durham DH1 3LE, U.K.

## Abstract

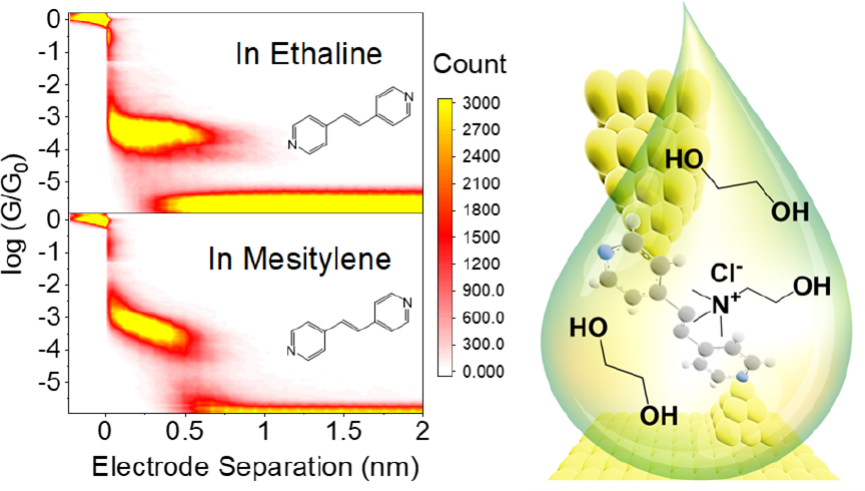

The environment surrounding a molecular junction affects its charge-transport properties and, therefore, must be chosen with care. In the case of measurements in liquid media, the solvent must provide good solvation, grant junction stability, and, in the case of electrolyte gating experiments, allow efficient electrical coupling to the gate electrodes through control of the electrical double layer. We evaluated in this study the deep eutectic solvent mixture (DES) ethaline, which is a mixture of choline chloride and ethylene glycol (1:2), for single-molecule junction fabrication with break-junction techniques. In ethaline, we were able to (i) measure challenging and poorly soluble molecular wires, exploiting the improved solvation capabilities offered by DESs, and (ii) efficiently apply an electrostatic gate able to modulate the conductance of the junction by approximately an order of magnitude within a ∼1 V potential window. The electrochemical gating results on a Au–**VDP**–Au junction follow exceptionally well the single-level modeling with strong gate coupling (where VDP is 1,2-di(pyridine-4-yl)ethene). Ethaline is also an ideal solvent for the measurement of very short molecular junctions, as it grants a greatly reduced snapback distance of the metallic electrodes upon point-contact rupture. Our work demonstrates that DESs are viable alternatives to often relatively expensive ionic liquids, offering good versatility for single-molecule electrical measurements.

## 1.0 Introduction

It is now possible to reliably form and measure the electrical characteristics of single-molecule junctions in a wide variety of solvents and electrolytes under a wide variety of experimental conditions. The electrical behavior of the junction can be recorded by using one of many techniques, such as scanning probe microscopy (SPM)^1,2^ and mechanically controllable break junctions (MCBJs).^3−5^ The versatility of these methods includes the ability to measure a wide variety of molecular targets under diverse conditions, with temperatures ranging from cryogenic to above ambient ones and environments spanning from UHV to solvents ranging from electrolytically nonconductive organic liquids to ionic liquids. There are a range of scanning-probe-based methods, which differ in how the junctions are formed but have the commonality of tethering a molecular target between a pair of electrodes to form a metal | molecule | metal junction. The most widely used method for recording single-molecule electrical characteristics is the scanning tunneling microscopy break junction (STM-BJ) technique.^6−8^ Such measurements are typically performed either in a solvent containing the molecular target or with the target formed as a self-assembled monolayer. The former “*in situ”* approach is often preferable over the “*ex situ*” self-assembly of molecular monolayers on substrates, in which case the solvent properties are very important. Advantages of the *in situ* approach over the *ex situ* approach can be that often the optimal conditions for the latter are unknown and might have to be optimized, while the *in situ* measurement directly from solutions of the target molecule removes this surface assembly preparation step. In addition, the *in situ* method provides a fresh supply of the molecular target from solution, as the junctions are continuously made, broken, and remade during the cyclic STM-BJ process.

An important consideration for the measurement liquid for two-terminal STM-BJ experiments is that it is a poor ionic (electrolytic) conductor so that leakage current is not an issue and the target molecule has sufficient solubility. Typical solution concentrations for such measurements are in the range of 10^–2^ to 10^–6^ M, depending on experimental requirements. The solvent may also be required to show a low vapor pressure to avoid evaporation and potential damage to instrumentation from solvent vapors in some scenarios. It is also desirable for the solvent system to have a low toxicity. Typical solvent systems used in the literature include mesitylene, 1,2,4-trichlorobenzene, propylene carbonate, 1,3,5-trimethylbenzene, and aqueous solution.^9−14^ Further requirements are added when experiments require electrochemical control. Here, the solvent requires sufficient ionic (electrolytic) conductivity, and in the case of the STM-based techniques, all but the very end of the STM tip needs to be covered with a wax or other insulating coating. This is to reduce tip leakage currents to low levels, which typically need to be much lower than setpoint tunneling currents. Aqueous electrolytes have been used, but these can be unsuitable where a wide electrode potential window is required. Ionic liquids have been used to greatly extend the electrode potential window.^13,14^ Using these, multiple redox switching events have been observed which are not accessible with aqueous electrolytes.^15,16^ Although ionic liquid deployment can be very effective, it does have some potential drawbacks. These include the fact that they can be relatively expensive or require specialist synthesis and/or purification. With this in mind, we evaluate here deep eutectic solvents as electrolytic media for single-molecule electronics studies.

Although the break-junction method can work in air, in liquid, and in vacuum, limited liquid media can be chosen for electrochemical STM (EC-STM) break-junction studies as highlighted above.^12−15,17−19^ This promoted our interest in extending the choices of electrolyte medium for EC-STM break-junction studies. There are several requirements for such candidates; they should (1) form an ionically conducting electrolytic solution; (2) have a wide electrochemical window for measurements; (3) be easily synthesized or purchased; (4) be cheap; (5) ideally be nontoxic and environmentally friendly. Based on the above criteria, water or aqueous electrolytes may seem to be the most obvious choice. However, in previous studies, it has been shown that aqueous electrolytes are often not the best suited medium for EC-STM break-junction studies, since the target molecule may be water insoluble. In some instances, this can be circumvented by preadsorption of the molecule on the substrate from a suitable solvent. This can be successfully implemented when the target molecules form self-assembled monolayers (SAMs), but this is not always straightforwardly the case. Perhaps more importantly, aqueous electrolytes provide a relatively narrow potential window, which may mean that the required potentials cannot be reached to observe either the redox transition or multiple redox states. For example, only one redox transition was observed for a pyrrolo-tetrathiafulvalene containing molecular bridge (pTTF) in aqueous electrolytes,^15^ while “soft gating” with a very broad peak was recorded for viologen-based junctions.^20^ By contrast, ionic liquids have been shown to perform well as solvent/electrolytes in both of these situations. Two separated transitions of a pyrrolo-tetrathiafulvalene containing molecular bridge were reported by the *in situ* break-junction method in ionic liquid, which contrasted with the single observed redox transition in aqueous electrolytes.^15^ In addition, the electrochemical conductance gating of a viologen-based junction in the ionic liquid 1-butyl-3-methylimidazolium triflate (BMIM-OTf) fitted the two-step (hopping) mechanism, exhibiting a “hard gating”, which contrasts with the broad conductance feature in aqueous electrolytes.^20^

Deep eutectic solvents (DESs) are a relatively new class of ionic liquid analogue that share many physical properties with ionic liquids.^21−24^ In addition, DESs can be relatively cheap and readily synthesized. DESs are eutectic mixtures composed of Lewis or Brønsted acids and bases with inherent ionic conductivity and good solubilizing properties, making them attractive electrolytes for electrochemistry. Despite the viscosity of the liquid resulting in a lower ionic conductivity, some low-viscosity DESs still have a conductivity on the order of tens of mS cm^–1^.^23^ Due to the large electrochemical window and acceptable ionic conductivity, DESs can display advantages over aqueous electrolytes and several nonaqueous solvents for electrochemical studies. There are now many examples in which DESs have been exploited for metal electrodeposition, electropolishing, and extraction.^21,23−25^ There are also a growing number of publications in which fundamental electrochemical behavior is examined in deep eutectic solvents, examples of which include diffusion coefficients of redox species,^26−28^ electron transfer kinetics,^27,29^ interfacial single-crystal electrochemistry,^30^ metal underpotential deposition,^31,32^ and the electrochemistry of graphene in DESs.^33^ Moreover, in a recent study, fast electron transfer (ET) has been observed in a typical DES, ethaline (1:2 choline chloride:ethylene glycol). The ET rate constants measured in ethaline are just slightly lower than that in acetonitrile for ferrocene and comparable with those in water for ferrocyanide.^29^ This provides an additional incentive for investigating DESs as alternatives to ionic liquids for single-molecular junction studies.

In this study, ethaline has been used with both the STM-BJ and EC-STM techniques as the solvent or electrolyte environment and has been shown to be an effective alternative to an ionic liquid for such measurements. Several two-terminal single-molecular junctions have been successfully measured by STM-BJ, including some molecules that show low solubility in common solvents and had not been successfully measured before. Conductance data from the molecular junctions have also been compared with those measured in a common nonaqueous solvent, mesitylene. Following this, the electrostatic (or electrolyte) gating of the molecular conductance of 1,2-di(pyridine-4-yl)ethene (**VDP**) in ethaline was examined within a bipotentiostatically controlled four-electrode cell. The conductance of **VDP** junctions varies as a function of the sample potential applied to the gold substrate electrode. This is consistent with previous findings employing aqueous HClO_4_ electrolytes.^12^

## 2.0 Experimental Details

In this study, a scanning tunneling microscope (STM) is used to form and break metal-to-metal contacts repeatedly between the STM tip and the substrate. A fresh Au–Au single-atom junction is formed when the STM tip is pushed into the substrate, followed by tip retraction to thin the Au–Au constriction down to a single atom (point contact with the quantum unit of conductance *G*_0_, 2*e*^2^/*h*; 7.75 × 10^–5^ S). With further tip retraction, the ultrathin gold metallic structure snaps back, and then, molecules can self-assemble in the nanogap to form a metal | molecule | metal junction. This results in an additional plateau in the current–distance plot. Otherwise, a rapid exponential decay is observed if no single molecular junction is formed. The STM-BJ system can be further adapted by adding extra electrodes for the electrochemical implementation (EC-STM). Here, a four-electrode bipotentiostat configuration applies independent electrode potentials to both the STM tip and the substrate.^18^ Data were acquired and processed using bespoke Python scripts and plotted using commercial software (Origin 2020b).

### 2.1 Ethaline Preparation

Choline chloride (ChCl) (>98.0%, Tokyo Chemical Industry UK Ltd.) was recrystallized from absolute ethanol, followed by filtration and drying under vacuum. ChCl was mixed with 2 equiv of ethylene glycol (99+ %, Merck) under N_2_ protection, and the mixture was heated with stirring at 100 °C until a homogeneous colorless eutectic liquid was formed. The liquid was then cooled at ambient temperature with a N_2_ balloon used to isolate the mixture from water absorption from the air.

### 2.2 Single-Molecule Conductance Measurements

The detailed experimental procedure is similar to that of our previous molecular conductance studies employing ionic liquids.^15^ The STM-BJ technique as described in the Introduction has been used to collect single-molecule current–distance (*I–s*) traces and with electrochemical control of single-molecule conductance versus electrode potential responses. In each cycle, the gold STM tip is crashed into the gold substrate and then retracted to form a single atomic point contact. Further retraction results in the metallic contact breaking and snapping back. Following this, a single molecule can then bridge into the freshly opened gap. Following further retraction, this molecular bridge can break, itself resulting in a current jump in the current–distance (*I–s*) trace. (Examples of traces can be found in the Supporting Information Figure S8). In the electrochemical STM (EC-STM) implementation, a four-electrode bipotentiostat configuration is added to the STM-BJ setup. This facilitates independent electrode potential control of both the tip and the substrate. To minimize the faradaic leakage currents, an additional requirement for this technique is the application of an insulating coating on all parts of the STM tip, except the apex, which is left uncovered. We used Apiezon for the tip coating, which was stable for the electrochemical STM-BJ data collected for VDP in ethaline (see later). A homemade Ag/AgCl electrode has been used with the EC-STM setup as the reference electrode, together with a Pt counter electrode and the insulated Au tip. To prepare a Ag/AgCl reference electrode, two silver wires were dipped into a 0.1 M HCl solution. One wire was connected to the positive terminal of a 1.5 V AA battery and the other to the negative end. After a few minutes, the positively polarized wire was coated with a uniform AgCl layer, while H_2_ gas bubbles evolved at the other electrode. Details can be found in the Supporting Information.^34^

## 3.0 Results and Discussion

### 3.1 Au–Au Snapback Distance

The STM-BJ method relies on the single-atom Au–Au junction being broken immediately prior to any molecular junction being formed. When the Au–Au contact breaks, the tip of the two gold electrodes relaxes, and a small gap (also called snapback distance) opens. The tip retraction continues, and the target molecule can bridge within this expanding gap until the resulting metal | molecule | metal junction cleaves. Therefore, the actual molecular junction length consists of two parts: the snapback distance and subsequent electrode separation of the retraction. The average snapback distance depends on the different solvent or electrolytic environments and ideally needs to be estimated for each one (selected typical values are 0.4 nm in mesitylene; 0.5 nm in a 1:4 THF:mesitylene mixture; 0.65 nm under ambient air conditions; 0.63 nm in ultrahigh vacuum).^35−38^ In this study, we find a snapback distance of 0.16 nm in ethaline in the absence of any target molecules, which is considerably shorter than for other media. Details of the calibration method can be found in the Supporting Information (Figure S9). The surprisingly short snapback distance holds great promise for small-molecule junction measurement.

### 3.2 Two-Terminal Junctions

**Figure 1:**
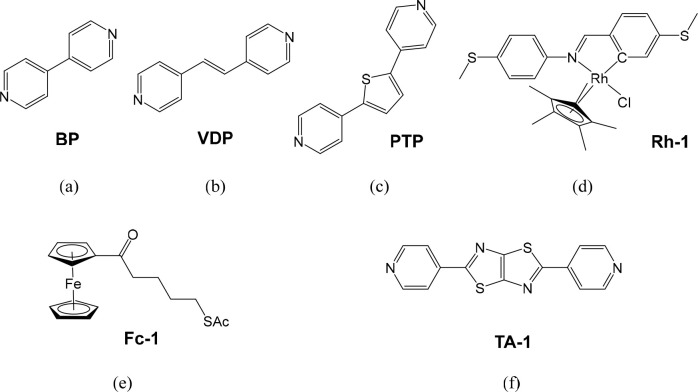
(a) 4,4′-Bipyridine (**BP**), (b) 1,2-di(pyridin-4-yl)ethene (**VDP**), (c) 2,5-di(pyridin-4-yl)thiophene (**PTP**), (d) rhodium complex (**Rh-1**), (e) ferrocene complex (**Fc-1**), (f) 2,5-di(pyridin-4-yl)thiazolo[5,4-*d*]thiazole (**TA-1**). In the main text, these compounds are referred to by their abbreviations in bold.

**Figure 2:**
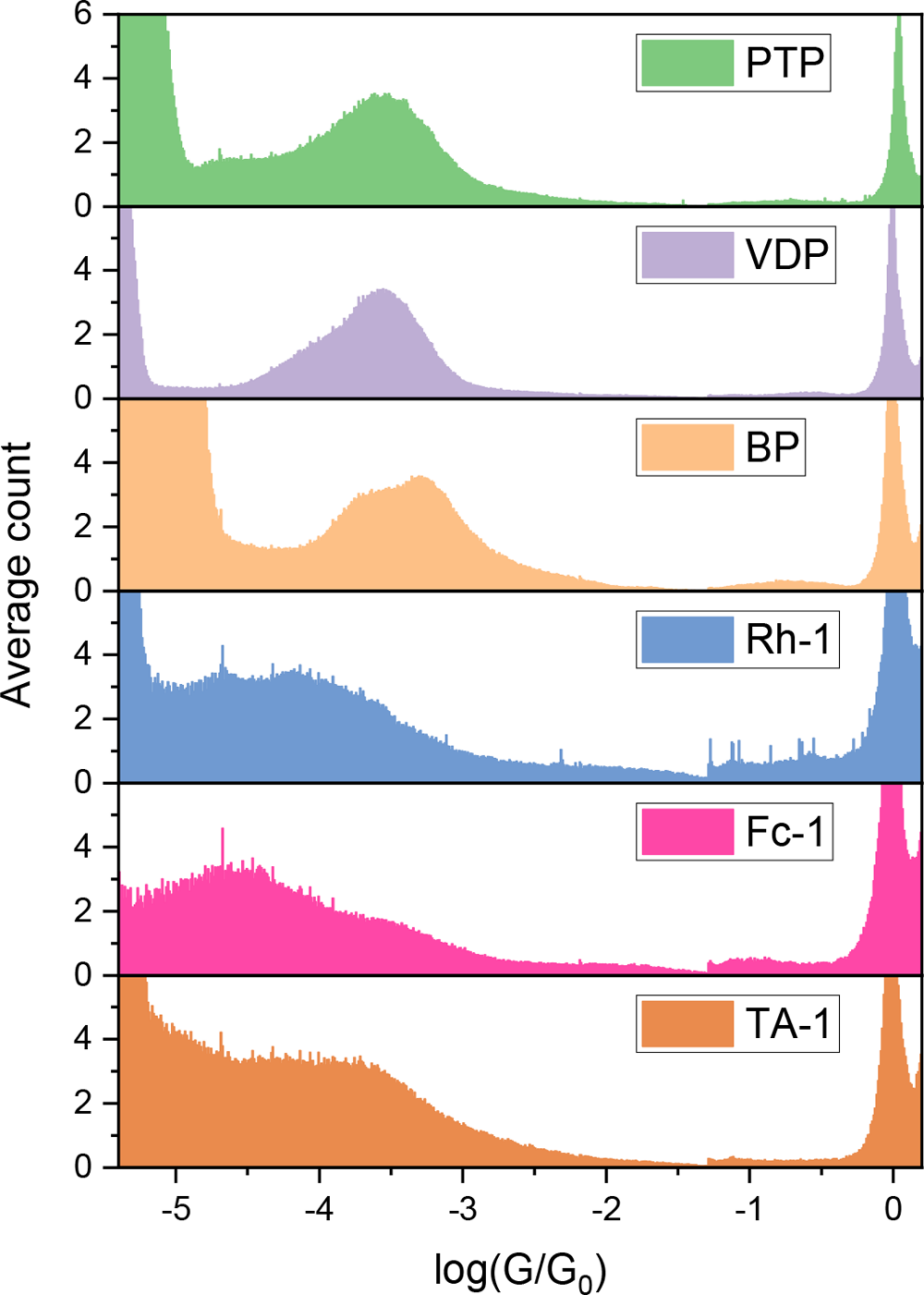
Conductance histograms recorded with the STM-BJ method for molecular junctions of the compounds shown in Figure 1, with Au electrodes, ethaline as the medium, and a bias voltage of 0.2 V. Details of each can be found in the Supporting Information Figure S10.

“Two-terminal junctions” refer to the conventional formation and measurement of metal | molecule | metal junctions, usually with Au–Au break junctions but in the absence of electrode potential control of the tip and substrate. Six two-terminal junctions were successfully measured in ethaline by STM-BJ (Figure 1). These range from the model pyridine-based targets (Figure 1(a–c)) to two metal complexes (Figure 1(d,e)) and a compound whose molecular conductance has never been successfully measured before because of its insolubility in the commonly used solvents for such measurements (Figure 1(f)). For these measurements, 1 mM solutions of each compound have been made in ethaline and evaluated at a fixed bias voltage of 0.2 V. Thousands of traces were collected for each compound and plotted with no further data selection into the conductance histograms (as log(*G*/*G*_0_)) for each sample (Figure 2). As is typical of the gold–pyridyl contact, two conductance features (high conductance, labeled HC, and low conductance, labeled LC) have been observed in most of the junctions, due to the conductance difference between two favorable geometrical configurations of these targets in the junction with different contacting between the pyridyl end groups and the gold contacts.^12,37^

By fitting each peak with Gaussian distributions, HC and LC peak values of all Au–molecule–Au junctions for the compounds in Figure 1 measured in ethaline are obtained (Supporting Information Table S2). The experimental molecular junction length and the calculated junction geometry (indicated by tilt angle α) for each junction are also listed. Despite the different solvent environments, values for Au–pyridyl compound–Au junctions (Figure 1(a–c)) are comparable with those recorded in other reports,^9,39,40^ thus, confirming ethaline as a suitable liquid medium for STM-BJ two-terminal measurements.

**Figure 3:**
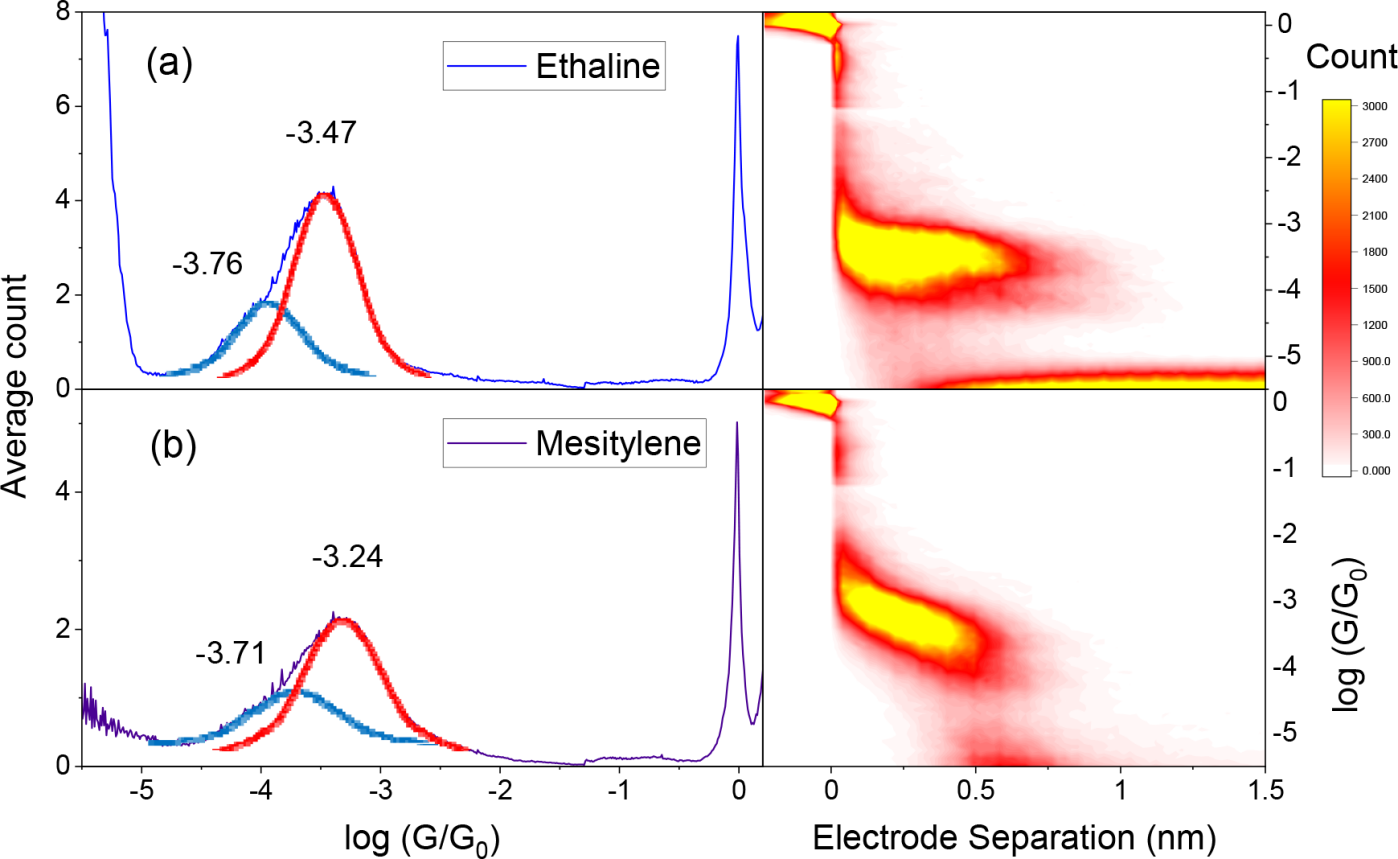
**VDP** junctions measured with the STM-BJ method in (a) ethaline (4766 traces) and (b) mesitylene (5391 traces) at 0.2 V bias voltage.

In addition, to highlight the advantages of measuring single-molecule junctions in ethaline compared to other commonly deployed solvents, both **VDP** and **PTP** junctions have also been measured in mesitylene (which alongside 1,2,4-trichlorobenzene is one of the most popular solvents used for STM-BJ measurements).^10,41−46^ When the HC and LC values of **VDP** in these respective liquids are compared (Figure 3 and Table S2 in the Supporting Information), the HC value measured in ethaline is slightly lower in conductance than that in mesitylene, while the LC values are similar in both solvents. Moreover, the slope (tilt) of the conductance plateau of the two-dimensional (2D) histogram (the right-hand side plots in Figure 3) recorded in ethaline environment is considerably flatter than that in mesitylene. This is a beneficial attribute regarding the formation of more defined molecular junctions, since it implies that there is much less conductance variation as the molecular junction is stretched. A similar behavior is also seen for PTP junctions (in the Supporting Information Figure S13 and Table S2), showing that the solvent type has a marked influence on the junction evolution during the stretching process. Tilted junction plateaus have been observed for conjugated molecular wires, and these have been related to a decreasing electrode–molecule coupling as the orientation of the molecular wire changes during junction stretching.^36,47^ Direct through-space tunneling between the electrode and the molecular wire π orbitals would be expected to be more pronounced for more tilted junction geometries. The differing solvation properties, molecular size, and viscosity might affect the junction formation and orientation in subtle ways, thereby impacting the tilt of the plateau in the 2D histograms. The solvating ability and viscosity of the DES may help to fix the molecule junction geometry as the tip retracts and may promote either more upright geometries or perturb the direct coupling between the molecule π-orbitals and the electrode. Evaluation of such a hypothesis would however require a high level of computational simulations to model the noncovalent interactions, the complex solvation, and the junction evolution and dynamics during the stretching, which are beyond the scope of this study.

Besides the model pyridyl molecular wires, **BP** and **VDP**, ethaline shows good solubility for several metal complexes and also target compounds for molecular conductance studies, which were previously found to be insoluble in solvents commonly used in STM-BJ studies. This includes **TA-1**, which has never been successfully measured before in other solvents, despite several attempts due to poor solubility. Although the compound was still not fully soluble in ethaline during preparation, reasonable conductance signatures could be clearly observed in the 2D histograms. This shows that ethaline provides additional opportunities for electrical conductance measurements for molecular wires, which have proven to be insoluble in organic solvents commonly used in STM-BJ studies.

### 3.3 Electrostatic Gating for VDP

**Figure 4:**
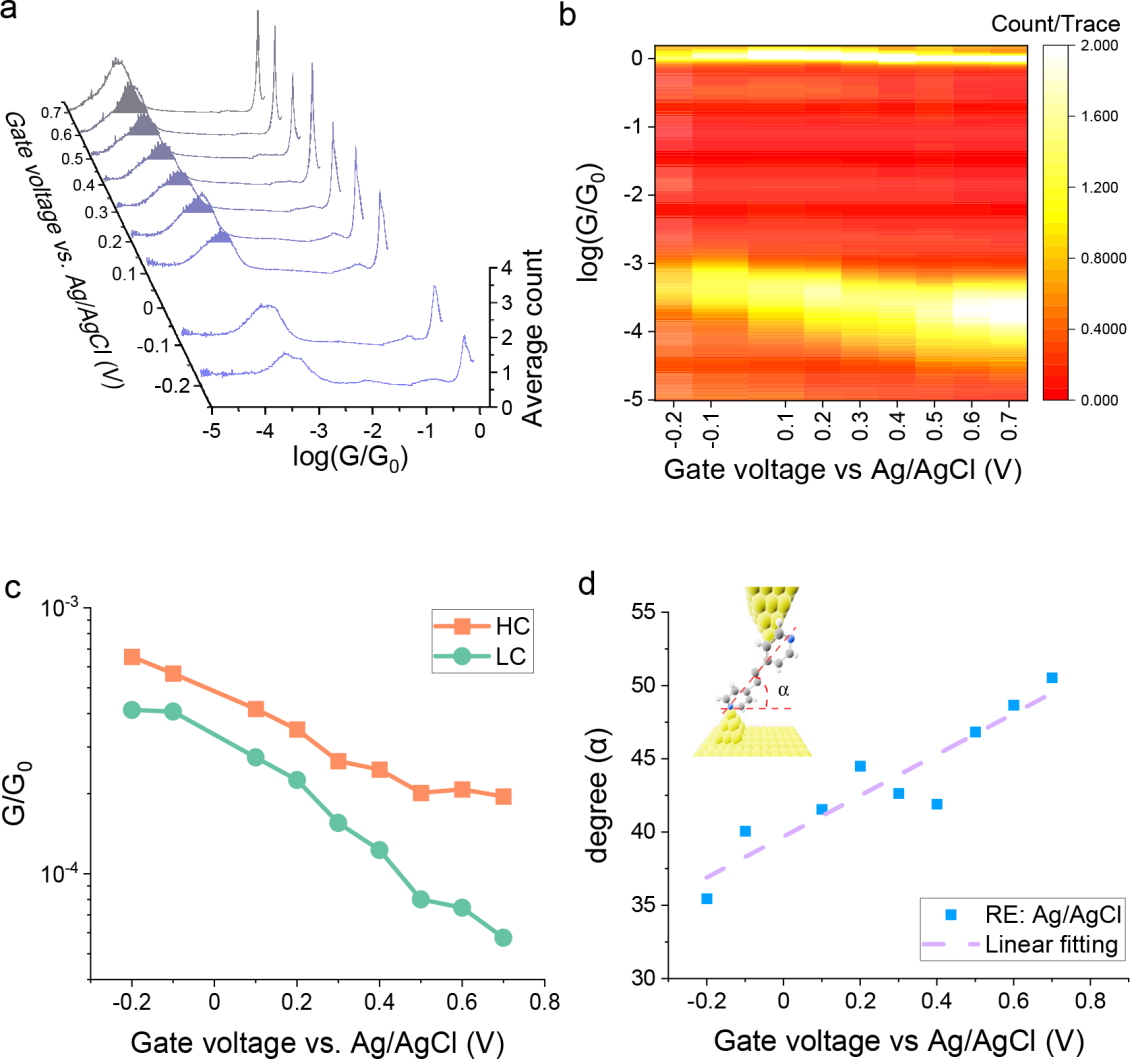
Data recorded using the STM-BJ method in ethaline under electrode potential control (EC-STM) with a Ag/AgCl reference electrode. (a) The histograms for **VDP** junctions in ethaline with different gate voltages. (b) The heatmap for the conductance of **VDP** junctions in ethaline versus gate voltage. (c) A plot of single-molecule conductance versus electrode potential for the HC and LC conductance values. A bias voltage of 0.1 V was used, and a Ag/AgCl electrode was employed as the reference electrode. (d) The change of the geometry (apparent tilt angle at the point for molecular junction cleavage) of the **VDP** junction vs different electrochemical gate voltages (electrode potentials), where the tilt angle α is defined as the angle between the molecular wire and the gold electrode surface (inset in Figure 4(d)). Data evaluated from 2D histograms to obtain the junction extension and thereby apparent tilt angle at the statistically determined point of molecular junction cleavage.

Figure 4a shows a set of histograms for Au–**VDP**–Au junctions with different electrode potentials applied to the gold substrate. These measurements were made with a Ag/AgCl reference electrode, which consisted of a Ag wire with an insoluble layer of AgCl, suitable for the EC-STM cell. The electrochemical gating voltage ranged from −0.2 to +0.7 V. Below −0.2 V, no junctions were observed, while the EC-STM system become very unstable when the sample electrode potential was set above +0.7 V. For each effective individual sample electrode potential, ∼4800 unselected traces were collected. Then, each set of data was plotted as a 1D histogram (Figure 4a). A corresponding conductance versus electrochemical gate voltage heat map is shown in Figure 4b, which illustrates the progression of single-molecule conductance to higher values with a more negative gate voltage. The mean conductance value for each peak in Figure 4a was fitted with a Gaussian distribution (using Origin 2020b software), and the trend is summarized in Figure 4c. Similar procedures have also been applied to the data measured with a Pt quasireference electrode (Figure S16). The data presented in the main paper were measured using a Ag/AgCl reference electrode, while complementary data with a Pt quasireference electrode are presented in the Supporting Information. These data sets are consistent, although the Ag/AgCl reference system is favorable, since it possesses greater stability (the electrode potential gating range for the Pt quasireference electrode is from −0.4 to +0.5 V). Detailed information concerning each sample potential with the two reference electrodes is summarized in Table S3.

In Figure 4, an extended electrochemical gating range (∼1 V) is recorded in ethaline, which is comparable with the previous studies in organic solvent^12^ or in aqueous phase.^13,14^ The advantage is that the electrolyte conductivity of ethaline is intrinsic without any requirement for the addition of a supporting electrolyte. The molecular conductance values shift higher when the sample electrode potential is adjusted to more negative values (Figures 4(c) and S3). The rate of change of conductance with electrode potential is different for the HC and LC peaks, and this could arise from different anchoring configurations yielding different coupling values in the single-level model (Breit-Wigner formula; see later). As discussed in Quek et al.,^37^ the low-conductance feature of 4-pyridyl junctions has both weaker coupling and better alignment to the electrode Fermi level, which would yield the higher “gating rate” we observed. Furthermore, a similar behavior for VDP has also been found by Brooke et al. in Ni–molecule–Ni junctions.^12^ In the absence of experimental values for gamma and epsilon for the low-*G* feature, we focus our later discussion on the high-*G* feature, where indeed we observe excellent agreement between our data and the single-level model.

As explained in previous works, the electrochemical gating effect can be attributed to the change in the electrode Fermi level (ε_F_) position relative to the molecular orbital caused by the potential applied between the electrode and the electrolyte solution.^12,48^ When the potential is moved to more negative values, ε_F_ is raised. Thus, the energy barrier between the Fermi level and the lowest unoccupied molecular orbital (LUMO) decreases, leading to an increase in conductance as pyridyl anchoring groups generally impose LUMO mediated tunneling. This is consistent with previous experimental results employing aqueous electrolyte gating.^12^

The increase in conductance may also be, in part, due to changes in junction geometry between the different gate voltages. Figure 4(d) shows that the apparent tilt angle of the VDP junction (determined at the end of the molecular plateau) becomes smaller when the electrode potential (“gate voltage”) moves to more negative values. Here, the tilt angle is defined as the angle between the VDP molecule and the surface of the gold electrode at the point where the molecular junctions are deemed to cleave (judged as at the end of conductance plateaus seen in 2D histograms). The smaller the tilt angle, the more tilted the molecular junction is with respect to the Au–Au contact axis. The tilt angle of the junction at each gate voltage with the Ag/AgCl reference electrode has been calculated and tabulated into Table S3 in the Supporting Information. Similar trends have also been observed in the relationship between the tilt angle vs gate voltage for equivalent data measured with a Pt quasireference electrode (Figure S17). When the geometry of the junction becomes more tilted, the nitrogen–gold bond tilts away from the plane of the pyridine ring, leading to an enhancement of the electronic coupling between the gold s-states and the LUMO π* orbital. This could then contribute to a conductance increase through the molecular junction.

**Figure 5:**
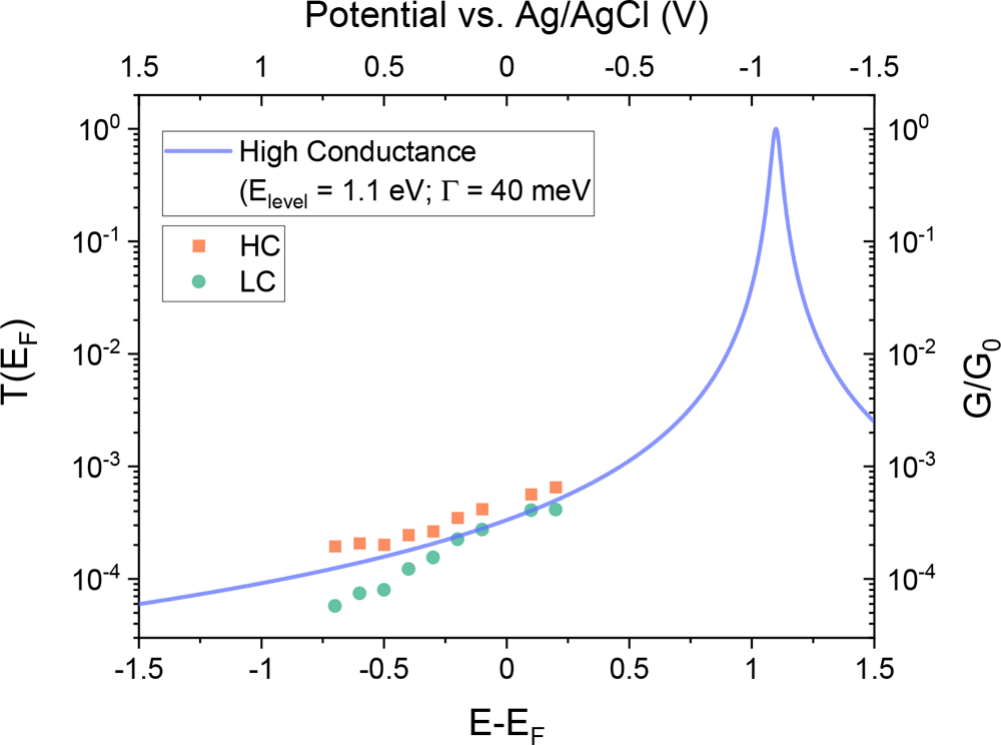
Experimental conductance values for the HC (red squares) and LC (green circles) and calculated *T*(*E*) for a Au–**VDP**–Au junction as a function of the applied electrochemical gating potential vs the Ag/AgCl reference electrode. The values quoted in the Figure for *E*_*Level*_ and Γ come from experimental data for reference.^49^

A fundamental classical model for phase coherent charge transport through a metal–molecule–metal junction involves a coherent transmission of electrons and holes from one electrode to the other through the molecular orbital that dominates transport. The transmission coefficient can be described using a Lorentzian form using the Breit–Wigner formula:^49−52^
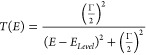
where *E*_*Level*_ is the alignment of the molecular orbital relative to the metal Fermi level and Γ is the broadening of the molecular orbital due to hybridization with the metal electrode. The junctions with Au electrodes have been well studied experimentally, and the transmission function has been well approximated by a single-Lorentzian form. It has been reported from experimental data that on average *E*_*Level*_ and Γ are, respectively, 1.1 eV and 40 meV for Au–VDP–Au junctions in the high-conductance state.^49^ It can be seen in Figure 5 that the experimental conductance for the electrochemically gated Au–VDP–Au junction (for data measured with a Ag/AgCl reference electrode) follows exceptionally well the single-level modeling, indicating a gate coupling coefficient close to unity (as it has been found for ionic liquids).^20^ This good correspondence between the experimental gating data for the Au–VDP–Au junction in ethaline electrolytes with the two-level phase coherent model verifies well the proposed mechanism of the electrostatic gating of VDP in ethaline.

## 4.0 Conclusions

In summary, we have employed a novel solvent medium for our STM-BJ/EC-STM system, and we have demonstrated that the deep eutectic solvent, ethaline, is a good alternative to ionic liquids for single molecular junction electrical measurements. This liquid is able to solvate two metal complexes and other previously insoluble molecular wire targets. Moreover, the gold snapback distance when the Au–Au junction breaks in DESs is shorter than that in other commonly used media for STM-BJ studies. 2D conductance histograms in DESs show flatter, better defined conductance plateaus, which are likely to result from the solvation of the molecular wire within the junction. We hypothesize that this might be a result of a reduction in through-space tunneling between the tilted molecular junctions and the contacting electrodes. This study also shows that ethaline is an exceptional medium for the measurement of single-molecule conductance under electrochemical conditions, with STM systems equipped with a four-electrode bipotentiostat control (EC-STM). Effective electrostatic gating with gate coupling close to unity can be achieved with this kind of solvent for the 1,2-di(pyridin-4-yl)ethene (**VDP**) system. Future work will focus on the evaluation of this solvent for the electrochemical gating of redox-active single-molecule junctions.

## Data Availability

STM-BJ data for all compounds discussed in this contribution are available under a CC-BY license in the University of Liverpool Data Catalogue at https://datacat.liverpool.ac.uk/2193/.

## Supporting Information Available

Methods; materials and synthetic procedures; details on STM-BJ and EC-STM measurements (PDF)
