# Supplementary material for: Single-Molecule Junction Formation in Deep Eutectic Solvents with Highly Effective Gate Coupling
Source: J Phys Chem C Nanomater Interfaces. 2023 Jun 27;127(26):12802–10. doi: 10.1021/acs.jpcc.3c03129 (PMC10331827; doi:10.1021/acs.jpcc.3c03129)
Supplement: Supplementary file 1 — jp3c03129_si_001.pdf [file jp3c03129_si_001.pdf]

Supporting information

Single-Molecule Junction Formation in Deep Eutectic Solvents with Highly Effective Gate Coupling

Xiaohang Qiao,[a] Andrea Vezzoli,[a] Shaun Smith,[a] Simon J. Higgins,[a] Ross J. Davidson,[b] Andrew Beeby[b] and Richard J. Nichols[a]

[a]*Department of Chemistry, University of Liverpool, Crown St, Liverpool, L69 7ZD, UK*

[b]*Department of Chemistry, Durham University, South Rd, Durham, DH1 3LE, UK*

Author Contributions

X.Q. Formal analysis: Lead; Investigation: Lead; Methodology: Supporting; Writing—Original Draft: Lead;

A.V. Formal analysis: Supporting; Funding acquisition: Supporting; Investigation: Supporting; Methodology: Supporting; Software: Lead; Supervision: Equal; Validation: Supporting; Writing—Review & Editing: Supporting.

S.S. Synthesis;

S.H. Formal analysis: Supporting; Validation: Supporting; Supervision: Equal.

R.D. Synthesis; Formal analysis: Supporting;

A.B. Synthesis; Formal analysis: Supporting;

R.N. Conceptualization: Lead; Data curation: Supporting; Formal analysis: Supporting; Funding acquisition: Lead; Investigation: Supporting; Methodology: Supporting; Supervision: Equal; Validation: Supporting; Writing— Review & Editing: Lead

Contents

1. Methods 4

2. Materials and synthetic procedures 4

2.1. Synthesis of reported compounds 5

2.2. NMR spectra of reported compounds 7

2.3. Crystallographic data 10

3. Details on STM-BJ and EC-STM Measurements 12

3.1. Determination of the snapback distance 13

3.2. Two-terminal STM-BJ junctions 14

3.2.1. Determination of the molecular junction length 16

3.2.2. Summary table of the 2-terminal junctions 17

3.2.3. Calculation of the tilt angle 18

3.2.4. Comparison of Au-PTP/VDP-Au junction in different liquid environment. 19

3.3. EC-STM on the Au-VDP-Au junctions 20

3.3.1. Summary table of the EC-STM Au-VDP-Au junctions 21

3.3.2. EC-STM Au-VDP-Au junctions with a Pt quasi reference electrode. 22

3.3.3. Relationship between the geometry of the junctions vs. different gate voltages. 23

3.3.4. Single-level modelling for the Au-VDP-Au junctions with a Pt quasi reference electrode. 24

3.4. The preparation of the Ag/AgCl reference electrode. 25

4. References 26

1. Methods

Junctions were fabricated using the STM-BJ technique with a modified Keysight 5500 electrochemistry STM.[1] All measurements were performed in 1 mM deep eutectic solvent (ethaline) solution. The ethaline is made from 1 equivalent recrystallized choline chloride mixed with 2 equivalents ethylene glycol under N_2 protection at 100 ℃ until a homogeneous colourless liquid was formed. Measurements were performed using an Au tip (99.99+%, Goodfellow Cambridge Ltd) insulated with wax (Apiezon Wax W40, M&I Materials Ltd). Substrates were prepared by eBeam evaporation of Au (99.99+%, Goodfellow Cambridge Ltd) on freshly cleaved muscovite mica (Agar Scientific Ltd). Conductance histograms were compiled from more than 4000 consecutively measured traces with no data selection.

2. Materials and synthetic procedures

Instrumentation. NMR spectra were recorded in deuterated solvent solutions on a Varian VNMRS-600 spectrometer and referenced against solvent resonances (^{1}H, ^{13}C). ASAP data were recorded on a Xevo QTOF (Waters) high-resolution accurate mass tandem mass spectrometer equipped with an atmospheric pressure gas chromatograph (APGC) and an atmospheric solids analysis probe (ASAP). MALDI data were recorded on a Bruker Autoflex II ToF/FoF spectrometer. Microanalyses were performed by Elemental Analysis Service, London Metropolitan University, UK, or Durham University Elemental analysis service. 4,4′-Bipyridine (**BP**) and (E)-1,2-di(pyridin-4-yl)ethene (**VDP**) were sourced from Sigma-Aldrich (now Merck), while 2,5-di(pyridin-4-yl)thiophene (**PTP**), $[Cp^*RhCl_2]_2$,[2] 1-(5-bromopentanoyl)ferrocene,[3] 4,7-di-2′-(5′-bromo)-thienyl-2,1,3-benzothiadiazole,[4] 2,5-di(pyridin-4-yl)thiazolo[5,4]thiazole[5] (**TA-1**) were prepared according to literature methods. For details of the atom labels, see the NMR spectra of the reported compounds.

2.1. Synthesis of reported compounds

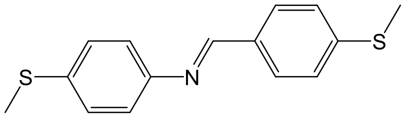

1-Bis(4-(methylthio)phenyl)methanimine (**L**H). 4-(Methylthio)aniline (0.89 mL, 1.00 g, 7.19 mmol) and 4-(methylthio)benzaldehyde (0.95 mL, 1.09 g, 7.19 mmol) were added to methanol (30 mL). The solution was stirred at room temperature for 2 hours, forming a yellow precipitate, which was collected by filtration and washed thoroughly with methanol to give the desired product. **Yield**: 1.78 g (91 %). ^{1}**H NMR** (600 MHz; CD_2Cl_2): δ_H 8.47 (s, 1H, H_d), 7.84 (d, $^3J_{HH}$ = 8.0 Hz, 2H, H_e), 7.37-7.30 (m, 4H, H_b+H_f), 7.22 (d, $^3J_{HH}$ = 8.0 Hz, 2H, H_c), 2.57 (s, 3H, H_g), 2.54 (s, 3H, H_a) ppm. ^{13}**C{^{1}H} NMR** (150 MHz; CD_2Cl_2): δ_C 158.7, 149.1, 143.3, 135.9, 132.9, 128.9, 127.4, 125.5, 121.5, 16.0, 14.8 ppm. **MS**(ASAP): m/z 274.1 $[M+H]^+$.

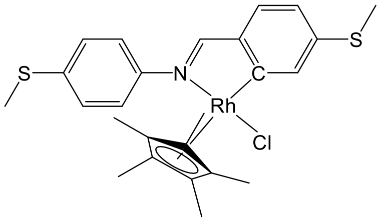

*RhCp*ClL* (**Rh-1**). $[Cp^*RhCl_2]_2$ (300 mg, 0.48 mmol) and $NaOAc{\cdot}3H_2O$ (331 mg, 2.43 mmol) were stirred in a MeOH:DCM (1:1, 30 mL) solution for 1 hour before **L**H (262 mg, 0.96 mmol) was added. The solution was stirred overnight at room temperature, then the solvent was removed under vacuum and the residue was passed through a silica plug using acetone as the eluent. The solvent was removed, leaving a red residue. Final purification was achieved by crystallisation from the evaporation of a MeOH:DCM (1:1) solution, producing red crystals. **Yield**: 320 mg (60 %). ^{1}**H NMR** (600 MHz; CD_2Cl_2): δ_H 8.12 (d, $^4J_{HH}$ = 3.7 Hz, 1H, H_g), 7.74 (br, 1H, H_d), 7.58 (d, $^3J_{HH}$ = 8.0 Hz, 2H, H_b), 7.47 (d, $^3J_{HH}$ = 7.9 Hz, 1H, H_f), 7.36 (d, $^3J_{HH}$ = 8.1 Hz, 2H, H_c), 6.99 (d, $^3J_{HH}$ = 7.9 Hz, 1H, H_e), 2.67 (s, 3H, H_a), 2.59 (s, 3H, H_h), 1.47 (s, 15H, H_i) ppm. ^{13}**C{^{1}H} NMR** (150 MHz; CD_2Cl_2): δ_C 185.9, 185.7, 170.9, 148.4, 143.5, 142.1, 137.3, 132.7, 128.8, 127.1, 122.6, 120.2, 96.3, 16.1, 15.4, 8.7 ppm. **MS**(MALDI): m/z 545.0 $[M]^+$, 510.0 $[M-Cl]^+$. **Anal. Calc.** for $C_{25}H_{29}ClNRhS_2{\cdot}$ ¼CH_2Cl_2: C, 53.47; H, 5.24; N, 2.47 %. **Found**: C, 53.40; H, 5.28; N, 2.54 %.

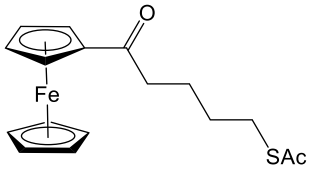

1-(5-Thioacetylpentanoyl)ferrocene (**Fc-1**). Potassium thioacetate (0.34 g, 3.00 mmol) was added to a solution of 1-(5-bromopentanoyl) ferrocene (1.00 g, 2.88 mmol) in anhydrous dimethyl formaldehyde (30 mL). The solution was stirred overnight at room temperature for 16 hours. The reaction mixture was extracted using diethyl ether and water, then the organic layer was collected, dried over $MgSO_4$, and filtered. The solvent was removed from the filtrate under *vaccuo* to give a red residue. Purification was achieved using silica chromatography eluted by a solvent gradient from neat hexane to neat DCM, to produce a red oil that solidified upon standing. **Yield**: 802 mg (81%). ^{1}**H NMR** (600 MHz; $CDCl_3$): δ_H 4.76 (t, $^3J_{HH}$ = 1.9 Hz, 2H, H_b), 4.48 (t, 2H, $^3J_{HH}$ = 1.9 Hz, H_c), 4.18 (s, 5H, H_a), 2.92 (t, $^3J_{HH}$ = 7.2 Hz, 2H, H_d), 2.71 (t, $^3J_{HH}$ = 7.2 Hz, 2H, H_e), 2.32 (s, 3H, H_h), 1.80-1.75 (m, 2H, H_f), 1.69-1.69 (m, 2H, H_g) ppm. ^{13}**C{^{1}H} NMR** (150 MHz; CD_2Cl_2): δ_C 203.9, 195.9, 78.9, 72.1, 69.7, 69.2, 38.9, 30.6, 29.2, 28.8, 23.4 ppm. **MS**(ASAP$^+$): m/z 345.050 $[M]^+$. **Anal. Calc.** for $C_{17}H_{20}FeO_2S$ ·¼H_2O: C, 58.55; H, 5.92 %. **Found**: C, 58.41; H, 5.93 %.

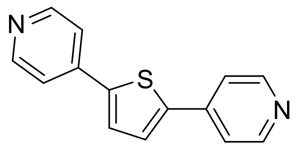

2,5-di(pyridine-4-yl)thiophene (**PTP**). A suspension of 4-pyridinylboronic acid (1.27 g, 10.32 mmol), 2,5-dibromothiophene (0.5 g, 2.06 mmol), $Pd(PPh_3)_4$ (0.238 g, 0.21 mmol) and potassium carbonate (2.9 g, 20.6 mmol) in degassed dioxane/water (15 mL / 5 mL) was refluxed for 48 hours under N_2 atmosphere. After cooling down to room temperature, the solvent was evaporated under reduced pressure and diluted with water. The resultant mixture was extracted with dichloromethane and the organic layer was dried over anhydrous magnesium sulfate and evaporated under reduced pressure. Purification by column chromatography over silica (100% ethyl acetate to 5% triethylamine in ethyl acetate gradient, Rf = 0.3), followed by recrystallisation from ethyl acetate (-18 °C) afforded the title compound as bright yellow solid (0.272 g, 55 %). ^{1}H NMR (500 MHz, $CDCl_3$) δ = 8.64 (d, $J_{H2'-H3'}$ = 4.2 Hz, 4H, $H^{3'}$), 7.53 (s, 2H, H^{2}), 7.51 (d, $J_{H2'-H3'}$ = 6.0 Hz, 4H, $H^{2'}$). ^{13}C NMR (126 MHz, $CDCl_3$) δ = 150.7 ($C^{3'}$), 142.8 (C^{1}), 140.9 ($C^{1'}$), 126.7 (C^{2}), 119.9 ($C^{2'}$). *m/z* (HRMS) *(CI)* 239.0652 $(M+H)^+$. $C_{14}H_{11}N_2S$ calc. 239.0643.

2.2. NMR spectra of reported compounds

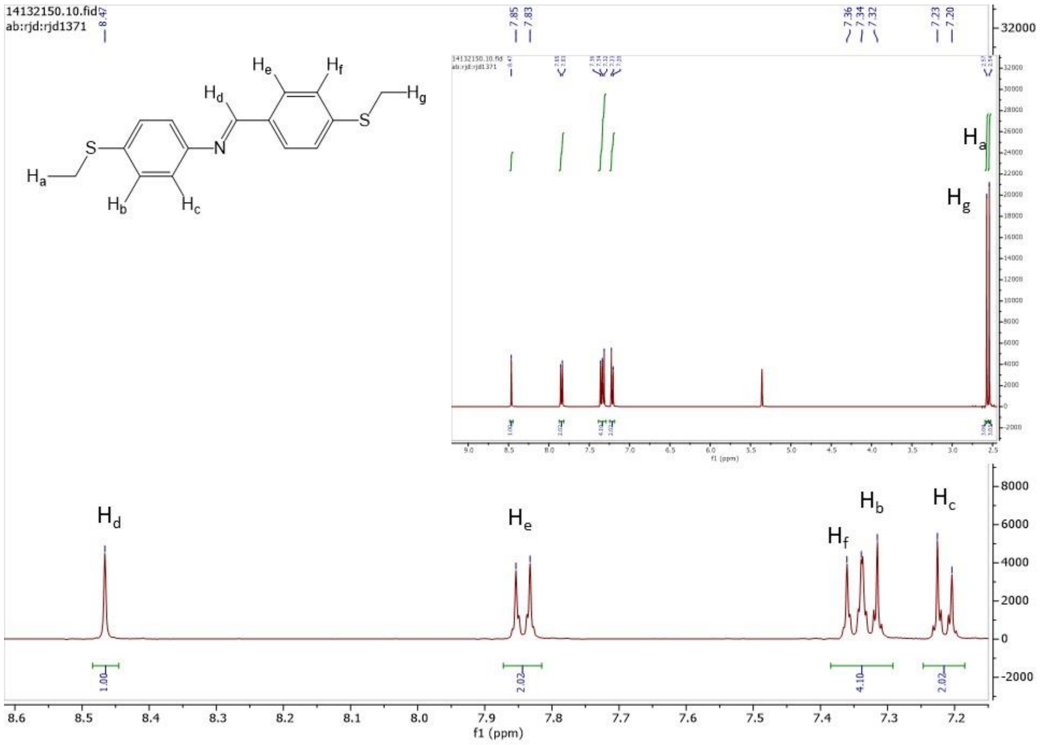

Figure S1. ^{1}H NMR spectrum of **L**H recorded in CD_2Cl_2.

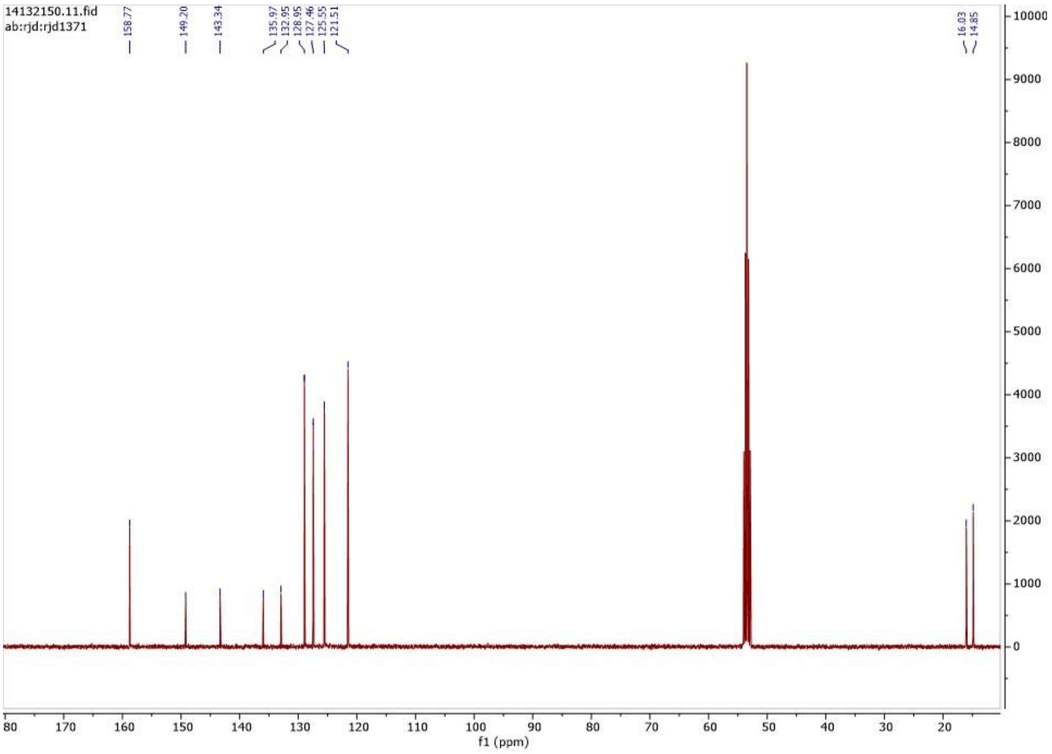

Figure S2. $^{13}C\{^{1}H\}$ NMR spectrum of **L**H recorded in CD_2Cl_2.

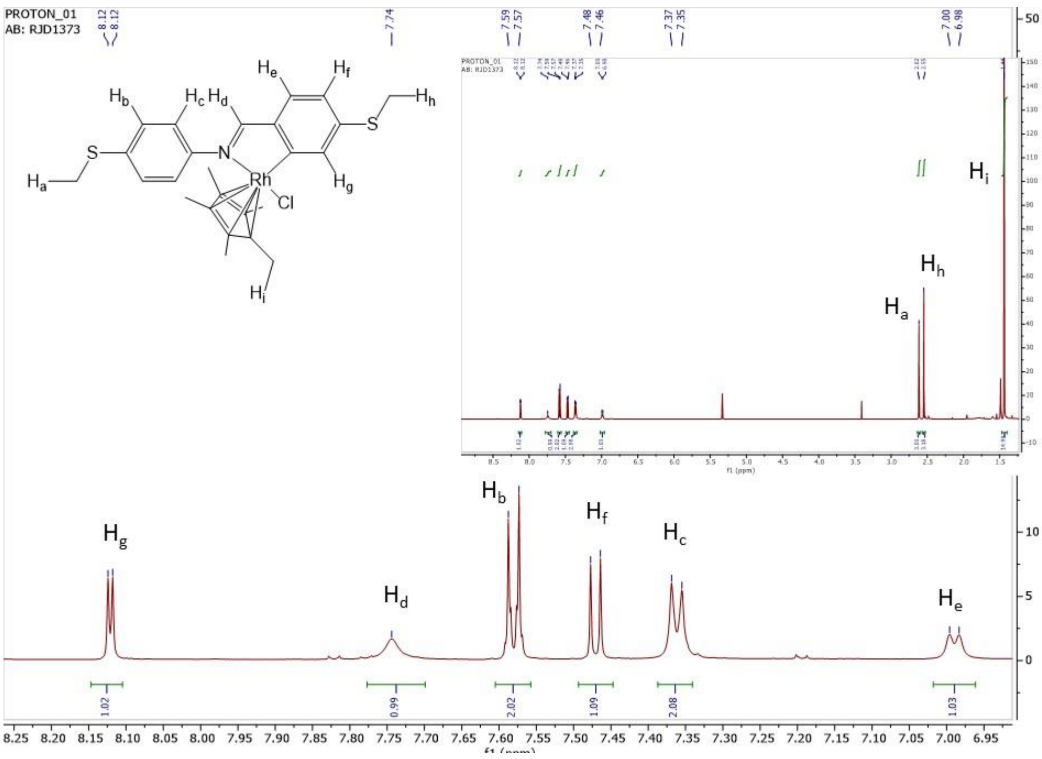

Figure S3. ^{1}H NMR spectrum of **Rh-1** recorded in CD_2Cl_2.

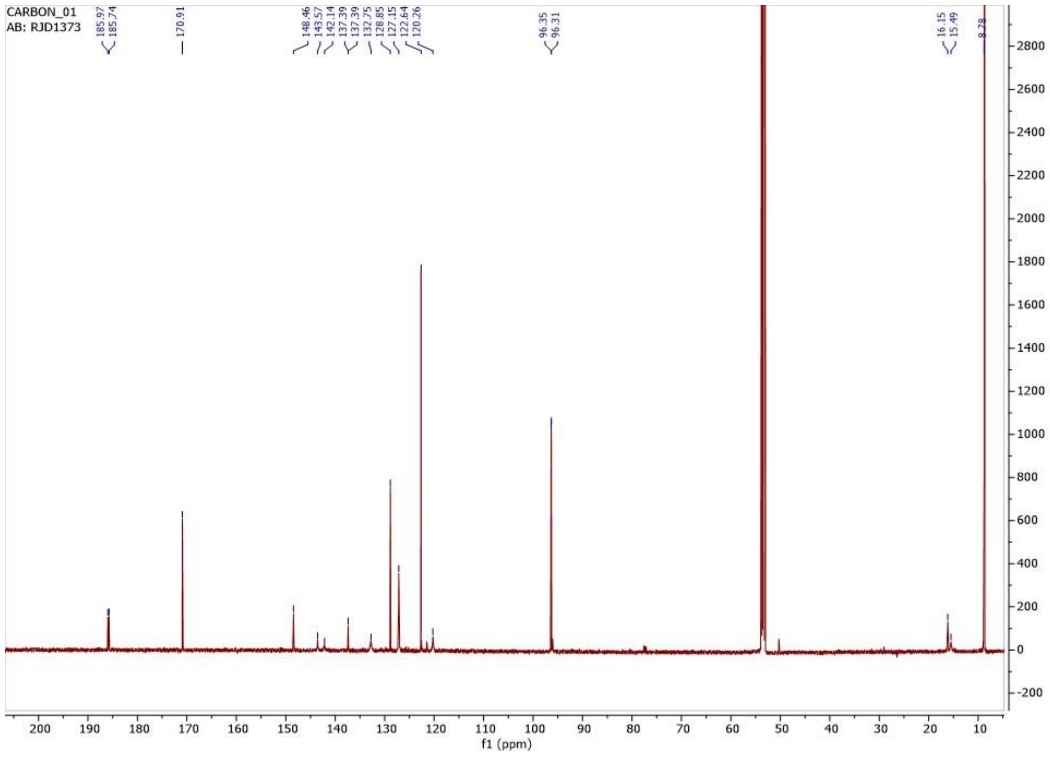

Figure S4. $^{13}C\{^{1}H\}$ NMR spectrum of **Rh-1** recorded in CD_2Cl_2.

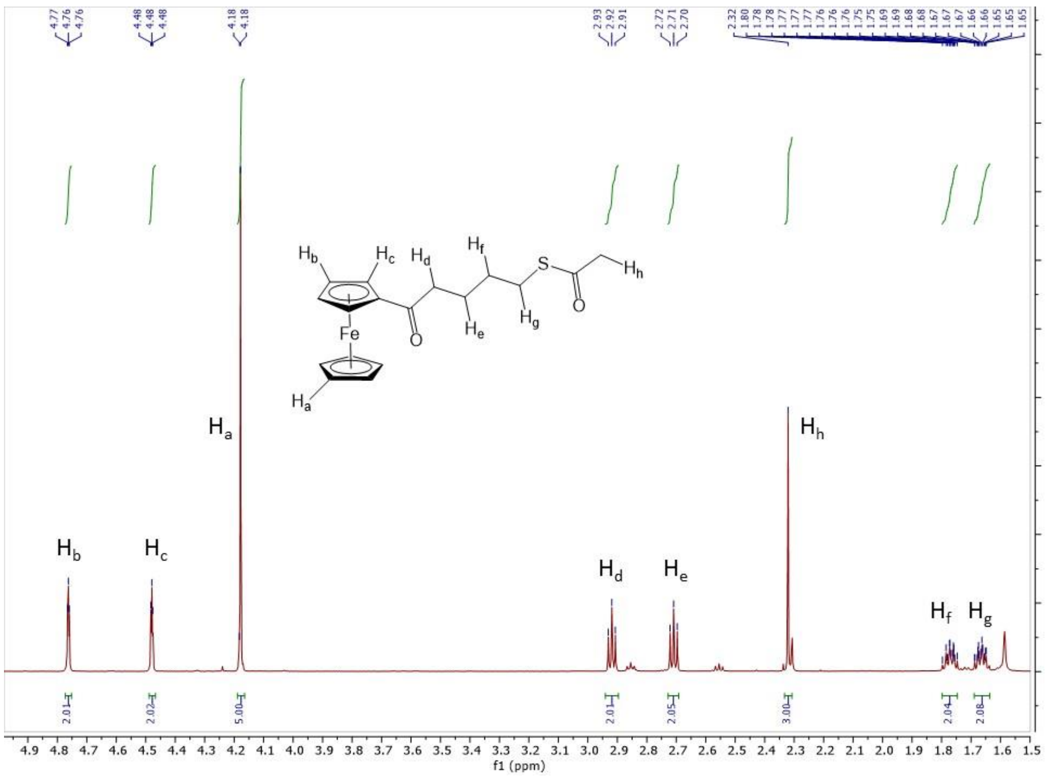

Figure S5. ^{1}H NMR spectrum of **Fc-1** recorded in $CDCl_3$.

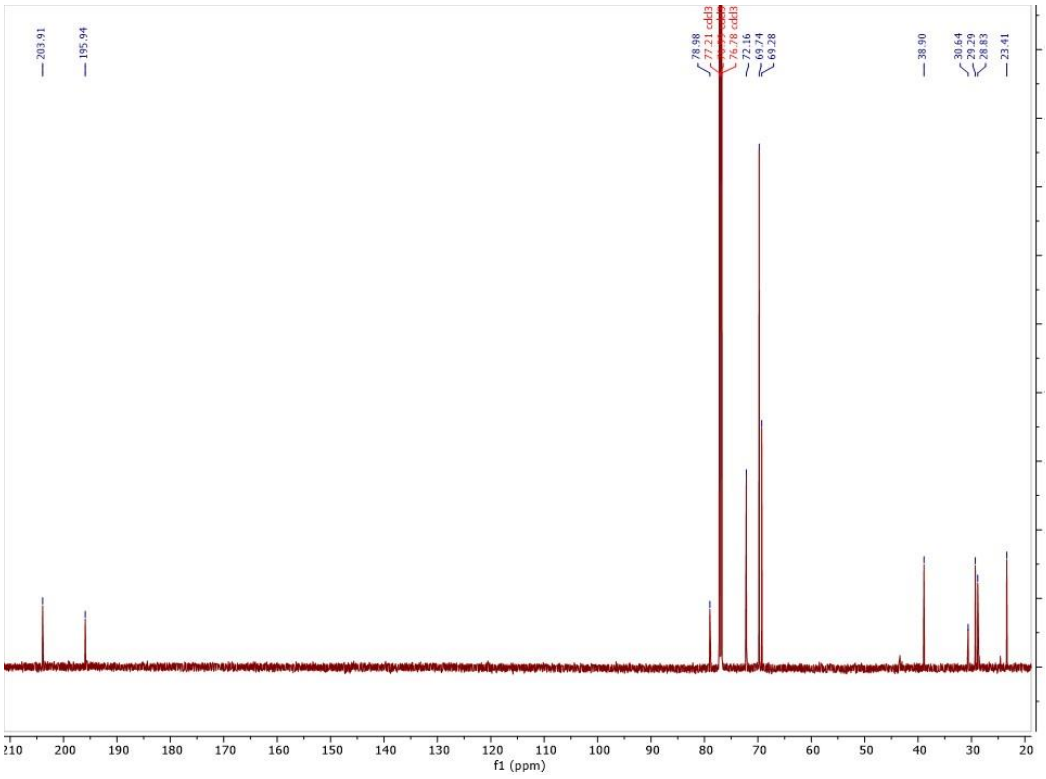

Figure S6. $^{13}C\{^1H\}$ NMR spectrum of **Fc-1** recorded in $CDCl_3$.

2.3. Crystallographic data

Identification code	15srv250
Empirical formula	$C_{25}H_{29}ClNRhS_2$
Formula weight	545.97
Temperature/K	120.0
Crystal system	triclinic
Space group	P-1
a/Å	8.5215(3)
b/Å	11.5488(4)
c/Å	12.3650(3)
α/°	93.340(2)
β/°	97.490(2)
γ/°	102.798(3)
Volume/Å^3	1171.73(6)
Z	2
ρ_{calc}g/cm^3	1.547
μ/mm^{-1}	1.034
F(000)	560.0
Crystal size/mm^3	0.18 × 0.09 × 0.06
Radiation	MoKα (λ = 0.71073)
2Θ range for data collection/°	4.7 to 58
Index ranges	$-11 \leq h \leq 11$, $-15 \leq k \leq 15$, $-16 \leq l \leq 16$
Reflections collected	16144
Independent reflections	6242 [R_{int} = 0.0649, R_{sigma} = 0.0935]
Data/restraints/parameters	6242/0/278
Goodness-of-fit on F^2	1.009
Final R indexes [I>=2σ (I)]	R_1 = 0.0513, wR_2 = 0.0900
Final R indexes [all data]	R_1 = 0.0813, wR_2 = 0.1019
Largest diff. peak/hole / e Å^{-3}	0.96/-0.63

Table S1. Crystal data and structure refinement for structures **Rh-1**.

The X-ray single crystal data for crystal **Rh-1** have been collected at temperature 120.0(2)K using MoKα radiation (λ =0.71073Å) on an Agilent XCalibur (Sapphire-3 CCD detector, fine-focus sealed tube, graphite monochromator) 4-circle κ-geometry diffractometer equipped with a Cryostream (Oxford Cryosystems) open-flow nitrogen cryostat. The structure was solved by direct method and refined by full-matrix least squares on F2 for all data using Olex26 and SHELXTL7 software. All non-hydrogen atoms were refined in anisotropic approximation, the hydrogen atoms were placed in the calculated positions and refined in riding mode. Crystal data and parameters of refinement are listed in **Table S1**. Crystallographic data for the structure have been deposited with the Cambridge Crystallographic Data Centre as supplementary publication CCDC-2217242.

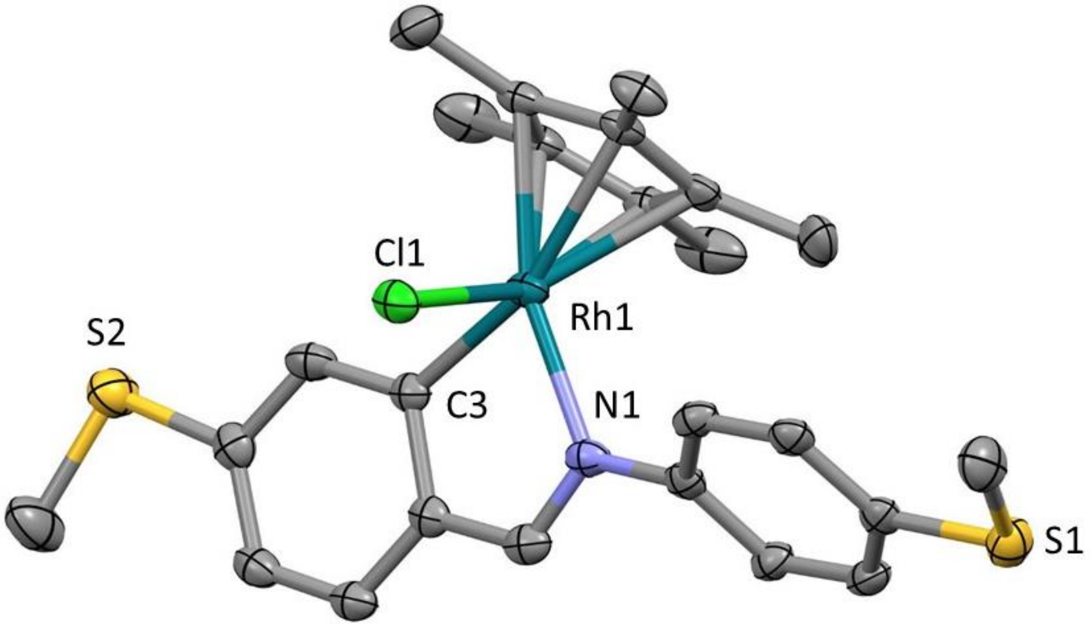

Figure S7. Crystal structure of **Rh-1**; hydrogen atoms removed for clarity; thermal ellipsoids displayed at 50% probability.

3. Details on STM-BJ and EC-STM Measurements

All the STM-BJ and EC-STM experiments were conducted on Keysight Technologies 5500 SPM, which is a proprietary STM, with modifications for our measurements based on the design from Meszaros et al.[8] The modified STM is furnished with a four-channel custom preamp and a National Instruments NI9215 USB data acquisition board (16- bit, 10 kSa/s). All 2-terminal junctions (gold STM tip and gold substrate contacts) were measured without electrochemical gate voltage. The normal STM-BJ cell kit consists of a 3D printed cell holder, a wax (Apiezon Wax W, M&I Materials) partially insulated STM tip (cut from the annealed 99.99+% Au wire, Goodfellow Cambridge Ltd) and a Au-on-mica substrate. The junctions were measured in 1 mM solution with ethaline with a bias voltage of 0.2 V.

A four-electrode cell is electrochemically controlled with a bipotentiostat (Keysight Technologies) for EC-STM experiments. The necessary two extra electrodes (a counter electrode: a coiled platinum wire, and a reference electrode: a self-made Ag/AgCl or a platinum wire as pseudo-reference electrode) have been integrated into the above STM-BJ cell kit for the EC-STM measurements. A constant bias (controlled by the bipotentiostat) has been applied between the insulated Au tip and the Au substrate. In addition, an electrochemical potential is applied between the Au substrate and the counter electrode, relative to the reference, and has been appropriately adjusted during the measurement as the electrochemical gate voltage. All EC-STM experiments have been measured in a 1 mM solution of **VDP** in ethaline at a bias of 0.2 V (sample positive).

For both STM-BJ and EC-STM technique, an insulated Au tip is required to minimize the leakage current (faradaic electrochemical current flow) from the Au tip. The leakage current for the insulated STM tips is monitored with the tip adjusted away from the surface of the gold substrate before the measurements are made. Good quality measurements can only be undertaken if this leakage current is much smaller than the molecular junction current. The partially insulated Au tip is driven towards the Au substrate to create a Au-molecule-Au junction having conductance $\gg G_0$, and then pulled back at a constant steady velocity of 20 nm/s. Each cycle results in a current versus tip-to-sample distance trace, and this is repeated continuously. Current is monitored during this continuous in and out cycling to accumulate thousands of traces for each 2-terminal junction determination or at each electrochemical potential. The conductance of the junction is then calculated as $G = I/V$ and quoted with respect to G_0. Results are compiled into the respective data plots (conductance histograms, heatmaps and density plots) shown in the main paper and here in the SI with no data selection.

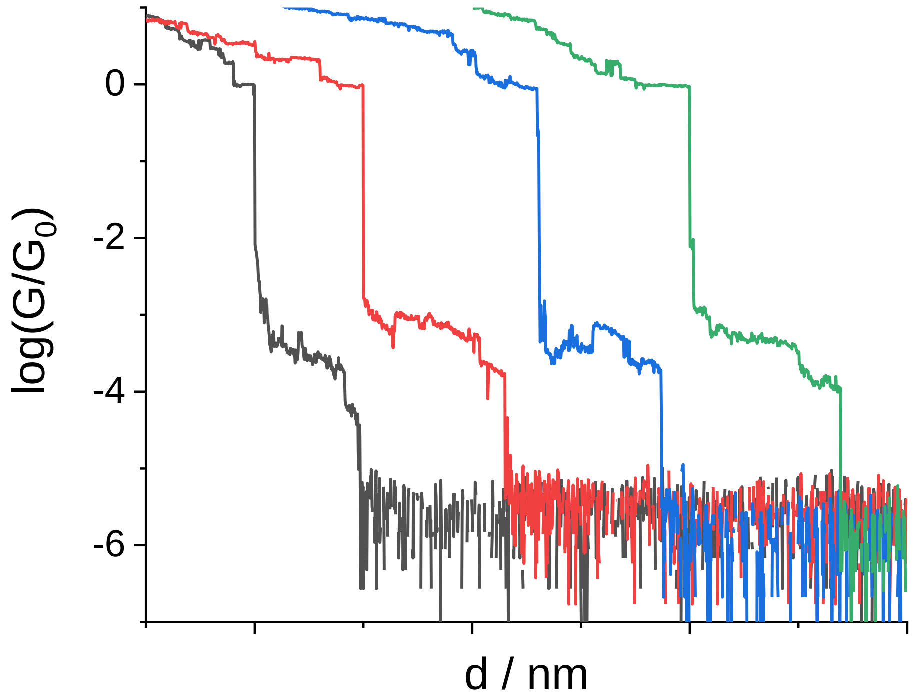

Figure S8: Examples of STM-BJ traces for **VDP** in ethaline at a bias voltage of 0.2 V.

3.1. Determination of the snapback distance

The snapback distance is the length between the two gold electrodes when the Au-Au single-atom junction breaks. It is surprisingly found that it is much shorter in ethaline than those measured in other common solvents mentioned in the paper. **Figure S9** shows an example conductance trace obtained from the STM-BJ measurements in pure ethaline without the presence of the molecular target.

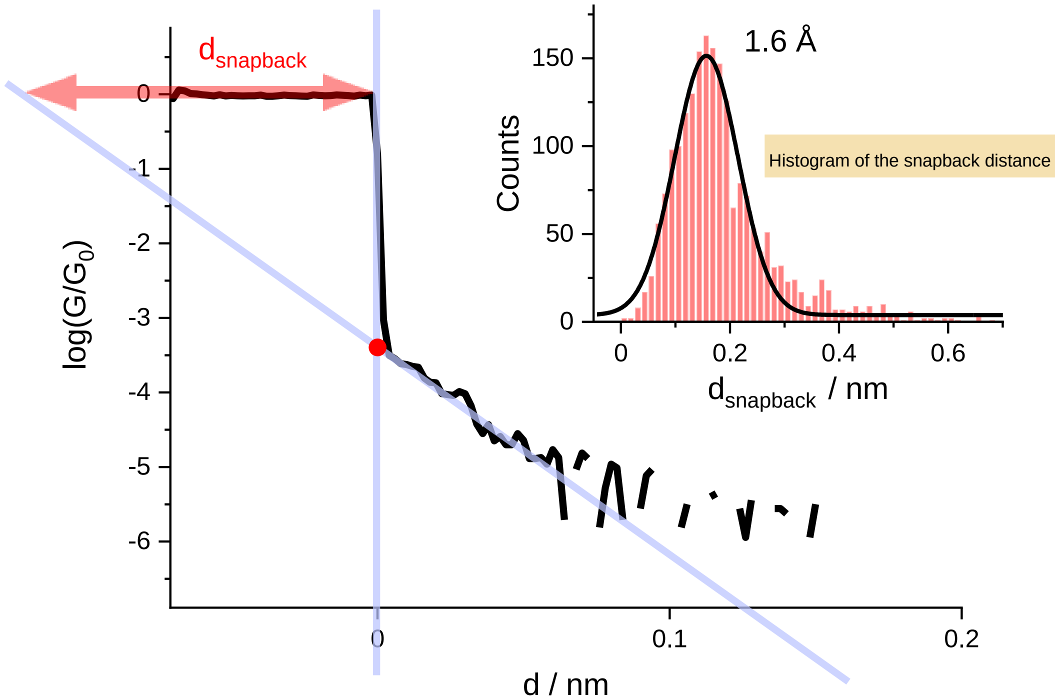

Figure S9. An example conductance traces from STM-BJ measurements performed in ethaline without the presence of any molecule and the explanation of snapback distance calculation. (Inserted) The histogram of the calculated snapback distance (calculated from 2031 traces).

These types of traces do not show the plateaux at $G \ll G_0$ attributed to the formation of molecular junctions. Instead, they show an exponential decay of the current because of the direct tunnelling between the electrodes when the Au-Au single-atom junction breaks. The Au atoms at the apex of the electrode experience restructuring and elastic relaxation after the atomic metal contact breaks resulting a rapid separation between the two electrodes. The size of the separation is called snapback distance and is calculated using a method similar to that used by Brooke et al.[9]. A linear regression line is fitted to the tunnelling current region of the trace, and then the snapback distance can be calculated by the intercept divided by the slope. The mean snapback distance was obtained from the histogram by fitting Gaussian distributions.

3.2. Two-terminal STM-BJ junctions

All histograms contain more than 4000 individual traces. The histograms are compiled with 100 bins per conductance decade and normalised as average counts (total counts/traces), while the heat maps are compiled with 20 bins per conductance decade and 50 bins per nanometre. The colour scale is normalised to the number of scans used to compile the map.

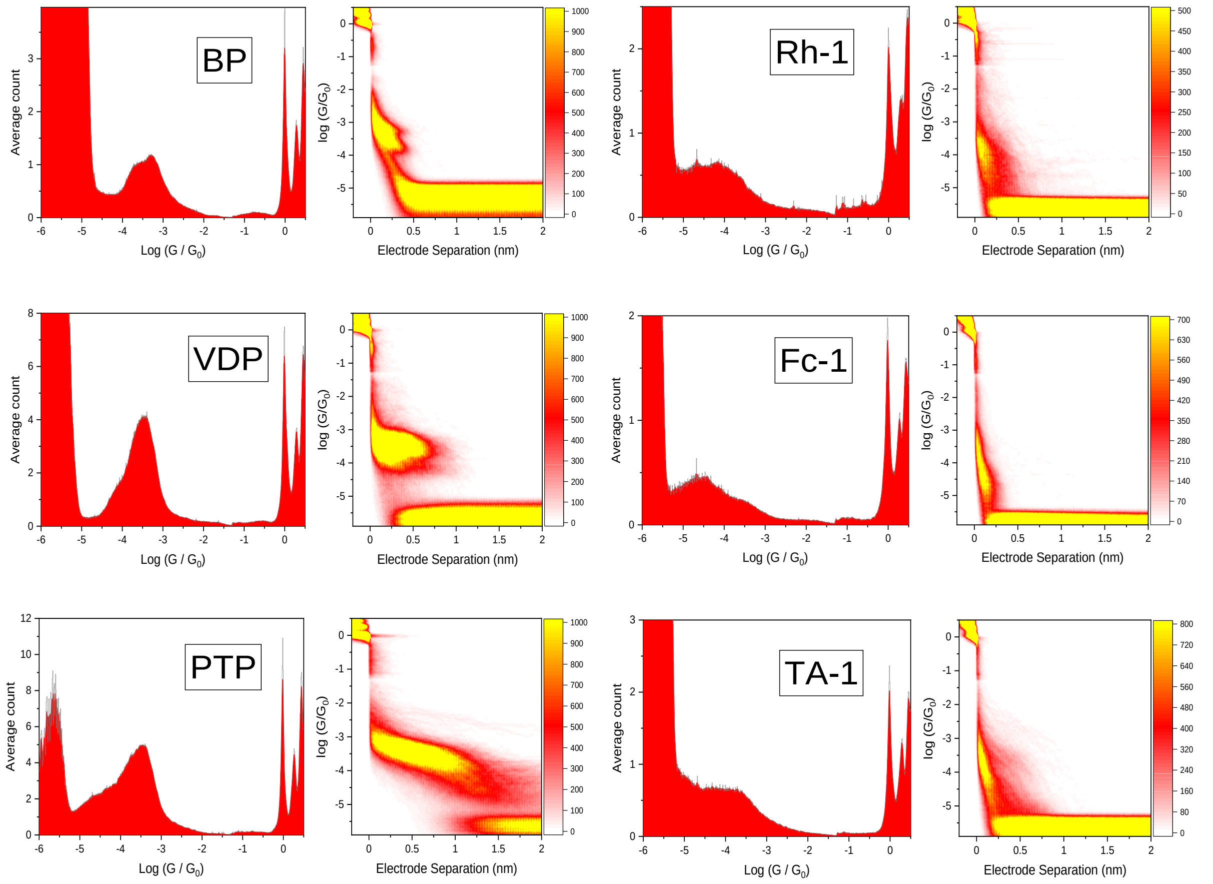

Figure S10. Raw conductance histograms and heatmap (2D conductance vs electrode withdrawal density map) for the compounds in **Figure 1** in the paper were recorded with the STM-BJ method technique, with Au electrodes, ethaline as the medium and a bias voltage of 0.2V.

The length of the electrode separation recorded by the STM was calculated by the 95th percentile of 2D conductance pattern. As illustrated by **Figure S11(a)**, a horizontal area (blue area) was selected and covers the range of the 2D conductance pattern (the banana shape with the red outline and yellow fill). The histogram of the electrode separation in the blue area and the 95th percentile was then calculated by the mean of the histogram plus twice of the standard deviation ($\mu+2\sigma$) through Origin. An example is plotted as **Figure S11(b)**.

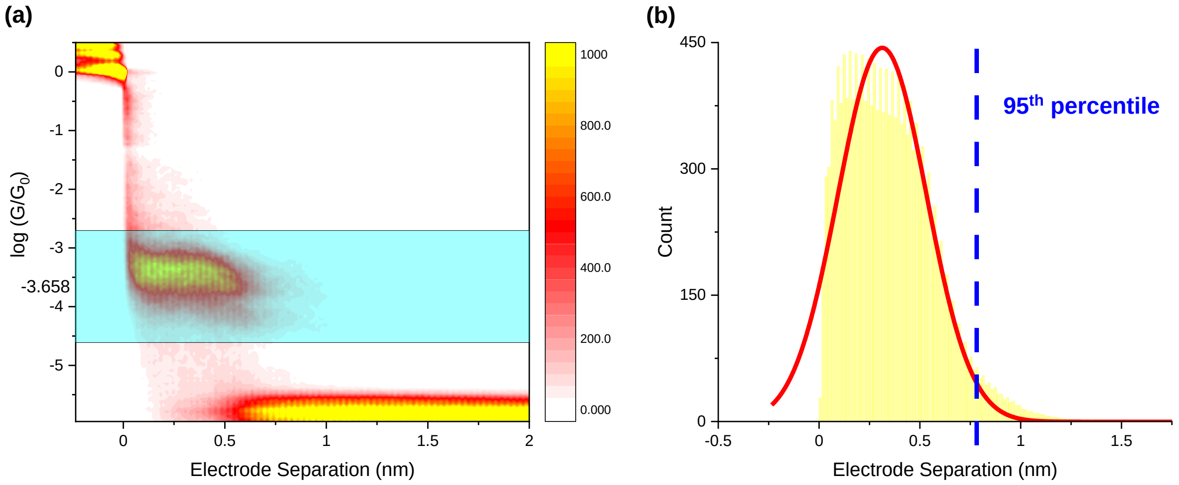

Figure S11. (a). An example of 2D histogram and the calculation area (blue) for determination of the 95th percentile of electrode separation recorded by the STM. (b). The 95th percentile of the length of the molecular junction.

3.2.1. Determination of the molecular junction length

Using the above method, the 95th percentile of each 2-terminal junction in **Figure 1** are summarized into the **Table S2**. The electrode separation recorded by STM are not the full junction length unless the snapback distance mentioned in **3.1** is added.

3.2.2. Summary table of the 2-terminal junctions

Along with the junction length, the conductance peak position (shown as $\log_{10}$ values) and the theoretical molecular length of each junction are also listed in the **Table S2**. Conductance peaks in the histograms were fitted with a gaussian distribution. The peaks with a higher conductance are labelled HC, and those with a lower conductance are labelled LC. The length of the molecules of each junction at their energy minimization were then calculated using the software Spartan 20V1.0.0.

Molecule	Peak position $\log(G/G_0)$	Molecular length calculated by Spartan (nm)	95th percentile of BJ electrode separation (nm)	Tilt angle (degree)
**BP**	HC: -3.30<br>LC: -3.60	0.71	0.54	50.0
**VDP**	HC: -3.47<br>LC: -3.76	0.94	0.76	76.4
**VDP (mesitylene)**	HC: -3.24<br>LC: -3.71	0.94	0.70	90.0*
**PTP**	HC: -3.48<br>LC: -4.52	1.07	1.34	90.0*
**PTP (mesitylene)**	HC: -3.43<br>LC: -4.57	1.07	1.07	90.0*
**Rh-1**	HC: -4.22<br>LC: -4.62	1.27	0.47	29.8
**Fc-1**	HC: -3.71<br>LC: -4.60	1.02	0.28	25.2
**TA-1**	HC: -3.92<br>LC: -5.05	1.30	0.77	45.6

Table S2. The conductance features of the molecules in **Figure 1** in the paper.

3.2.3. Calculation of the tilt angle

Figure S12 shows a simple illustration of a molecular junction illustrating the tilt angle of the molecular bridge. Here one anchoring group of the molecule directly attaches on the gold substrate. The other end can connect with the gold tip in two ways: (1) from the top (the red arrow) or (2) from the side (the blue arrow). There is an angle of inclination ($0 \leq \alpha \leq 90^{\circ}$) of the molecular junction. In this model α only reaches 90° when the gold tip is connected at the top position.

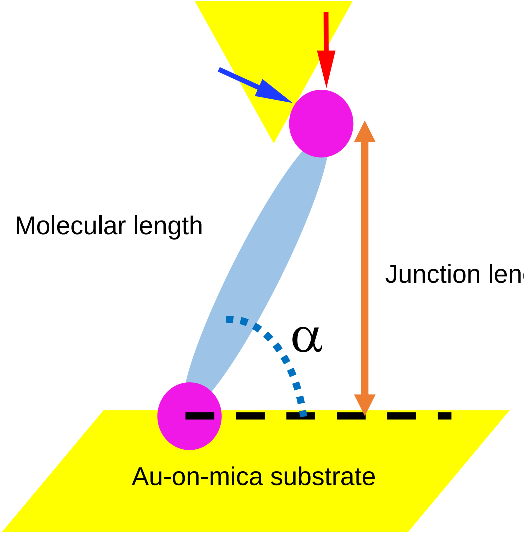

Figure S12. The schematic diagram of a molecular junction and the tilt angle α of the molecule.

The tilt angle for each junction listed in **Table S2** was derived from the arcsine of the junction length divided by the molecular length. α is smaller in the **BP** junction than the **VDP** junction due to a shorter molecular length, while α can be even smaller if the gold tip captures the molecule from the middle (i.e., Rh-1, Fc-1 and TA-1). The angles listed as 90.0^{0} in **Table S2** do not actually refer to the top connection, since the junction length with addition of the snapback distance is longer than the theoretical molecular length. A plausible reason for this could be that in some traces measured the molecules stack together through intermolecular interactions. In other words, in such cases there may be a mixture between single molecular junctions and multi molecular ones, which makes the determined junction length longer than that of a single molecule.

3.2.4. Comparison of Au-PTP/VDP-Au junction in different liquid environment.

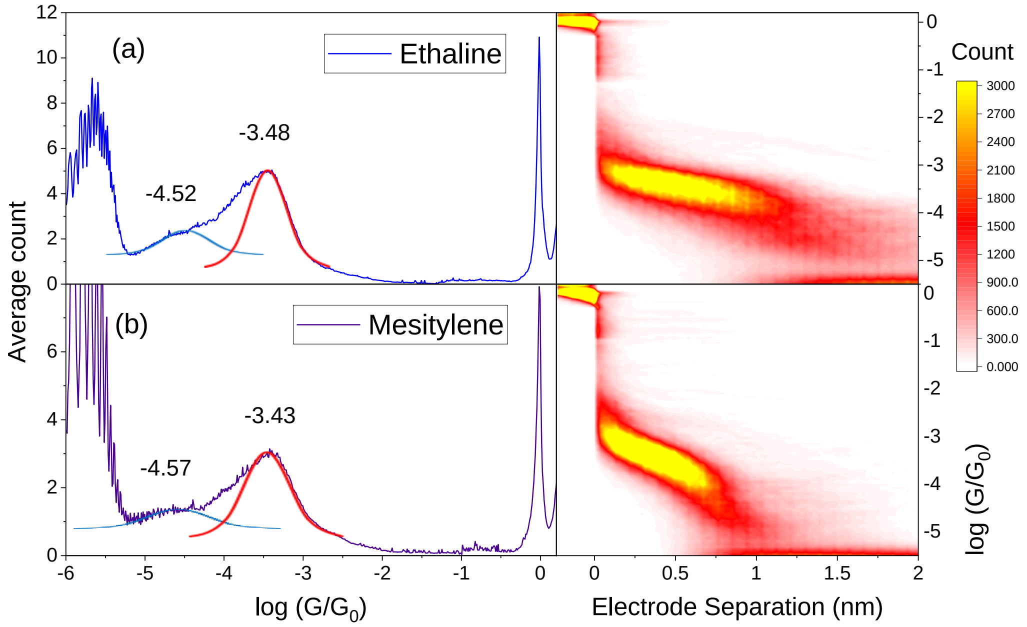

Figure S13. The STM-BJ data for **PTP**: (a) in ethaline (4327 traces) and (b) in mesitylene (4784 traces) in the bias 0.2V.

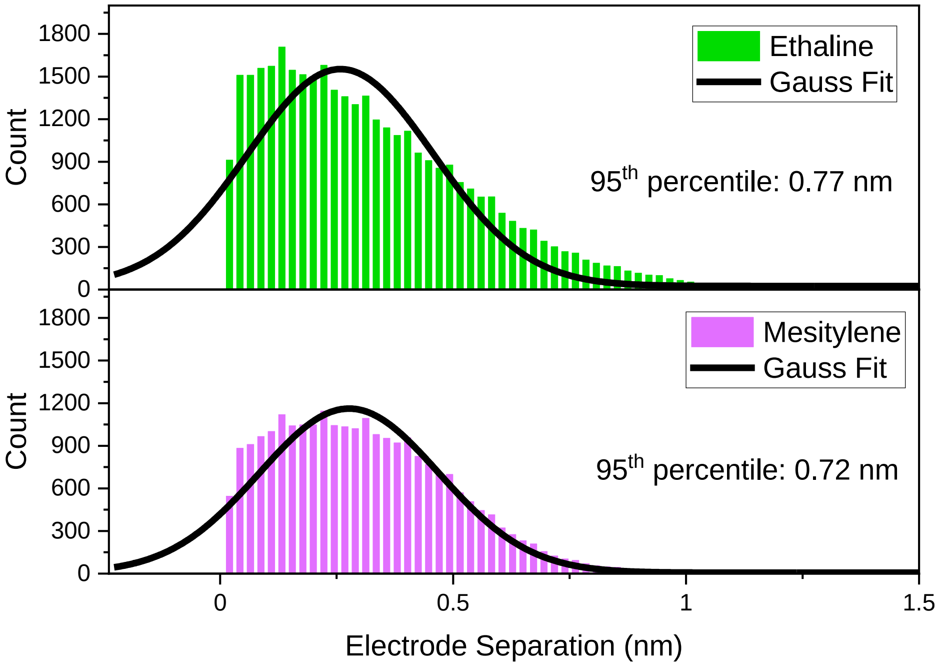

Figure S14: Break off distance histograms calculated from the 2D heatmap by placing a box enclosing the conductance plateau of **VDP** junctions in ethaline (upper) and mesitylene (lower) at a bias voltage of 0.2 V.

3.3. EC-STM on the Au-VDP-Au junctions

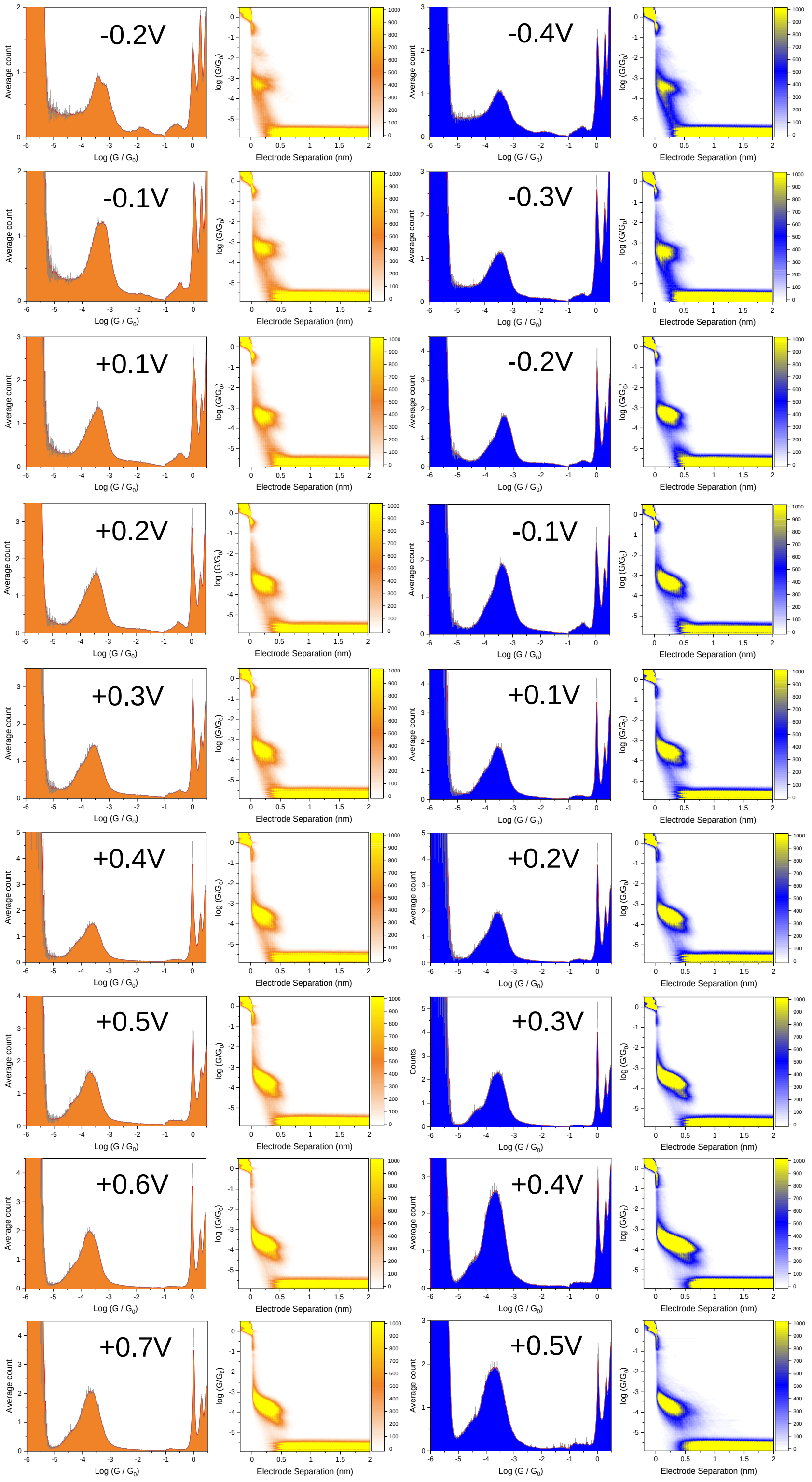

Figure S15. Raw conductance histograms and heatmaps (2D conductance vs electrode withdrawal density map) for **VDP** in **Figure 1** in the paper. Data recorded with the EC-STM technique, with Au electrodes, Ag/AgCl (orange) and Pt (blue) reference electrodes, ethaline as the medium and a fixed tip-sample bias voltage of 0.1 V. All histograms contain more than 4000 individual traces.

All the histograms and 2D conductance versus electrode withdrawal density maps of the EC-STM Au-**VDP**-Au junction at a fixed bias voltage of 0.1V and various gate voltage under electrochemical control with Ag/AgCl and Pt reference electrode are shown above (**Figure S15**). All histograms contain more than 4000 individual traces. The histograms were compiled with 100 bins per conductance decade and normalised as average counts (total counts/traces), while the heat maps were compiled with 20 bins per conductance decade and 50 bins per nanometre. Colour scale normalised to the number of scans used to compile the map.

3.3.1. Summary table of the EC-STM Au-VDP-Au junctions

Potential (mV)	VDP junctions with Ag/AgCl electrode			VDP junctions with Pt electrode		
	HC $\log(G/G_0)$	LC $\log(G/G_0)$	Tilt angle (degree)	HC $\log(G/G_0)$	LC $\log(G/G_0)$	Tilt angle (degree)
**-400**				-3.36	-3.50	38.3
**-300**				-3.42	-3.58	38.1
**-200**	-3.18	-3.38	35.5	-3.33	-3.68	43.9
**-100**	-3.25	-3.39	40.1	-3.38	-3.79	49.8
**0**						
**100**	-3.38	-3.56	41.6	-3.54	-3.95	47.9
**200**	-3.46	-3.65	44.5	-3.59	-4.17	51.0
**300**	-3.58	-3.81	42.6	-3.61	-4.29	56.1
**400**	-3.61	-3.91	41.9	-3.70	-4.40	79.5
**500**	-3.70	-4.10	46.8	-3.73	-4.43	55.4
**600**	-3.68	-4.13	48.7			
**700**	-3.71	-4.24	50.5			

Table S3. The conductance peak position of **VDP** junction and the tilt angle of the junction at each gate voltage with Ag/AgCl and Pt reference electrodes, respectively. Values for the HC and LC conductance peaks (shown as $\log_{10}$ values) refer to the high conductance peak and low conductance peak, respectively, and the "tilt angle" refers to the angle between the molecule and the surface of the gold electrode, judged at the end of the plateau (α in **Figure S12**). The calculation method of the conductance peak position and the tilt angle here is the same as for **Table S2**.

3.3.2. EC-STM Au-VDP-Au junctions with a Pt quasi reference electrode.

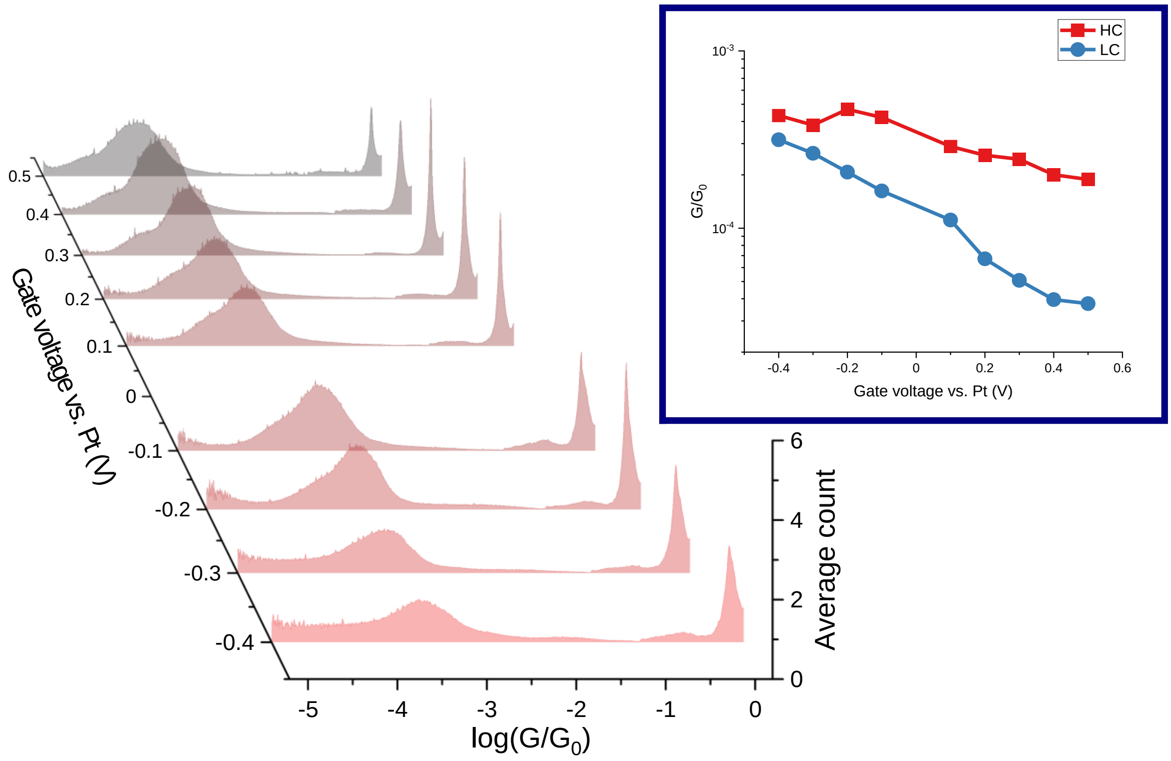

Figure S16. Conductance histograms for **VDP** junctions in ethaline for different sample electrode potential values. A Pt electrode was used as a quasi-reference electrode here.

There is a drop (or a plateau is reached) in the measured conductance values for HC peak using a Pt reference electrode when the sample potential voltage is set more negative than -0.2 V, which may arise from less stability or non-linearity with this quasi-reference electrode system as it is not apparent in the Ag/AgCl reference electrode data.

3.3.3. Relationship between the geometry of the junctions vs. different gate voltages.

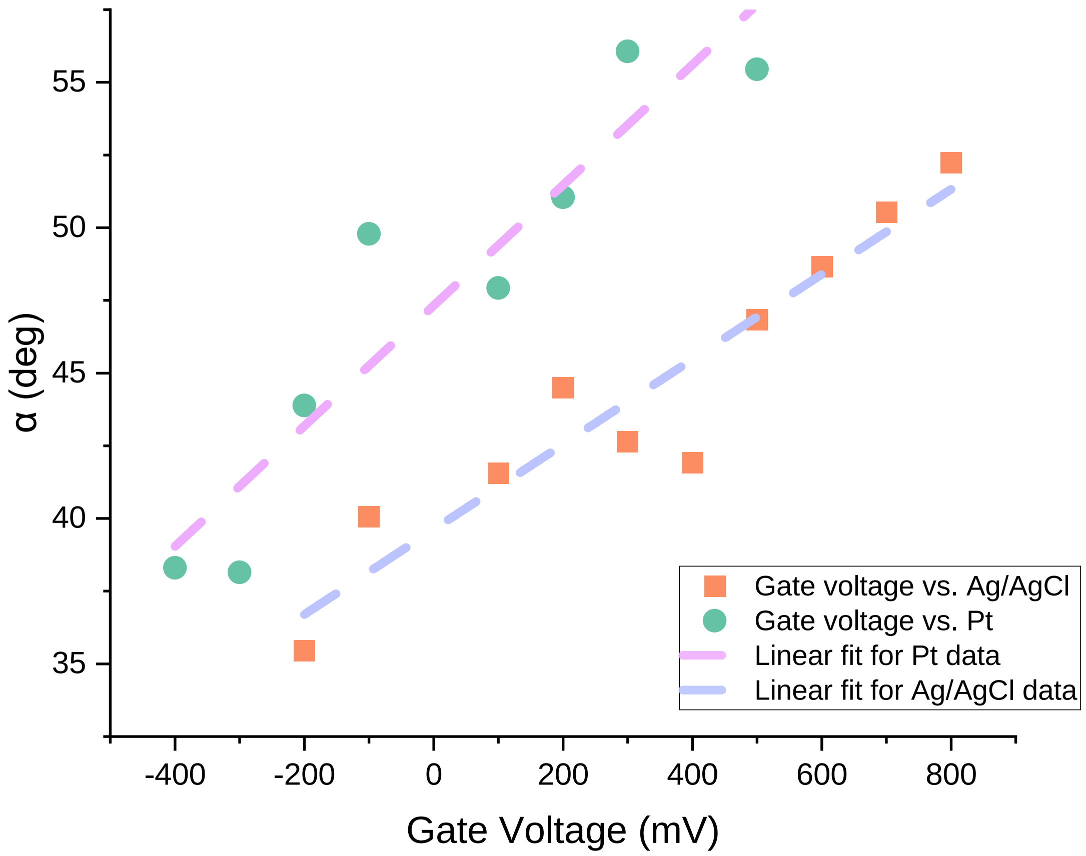

Figure S17. The trend of the geometry of the junction (with the tilt angle measured in the method discussed in **Figure S12**) measured with Pt (green points) and Ag/AgCl (orange points) reference electrodes vs. different gate voltages. Best linear fits to these data are shown as purple dashed and blue dashed line respectively. More negative gate voltage leads to a smaller tilt angle, which means the junction becomes more tilted with respect to a projected line between the two gold contacts. The difference between the two dataset is attributed to the lower stability of the Pt quasi reference electrode.

3.3.4. Single-level modelling for the Au-VDP-Au junctions with a Pt quasi reference electrode.

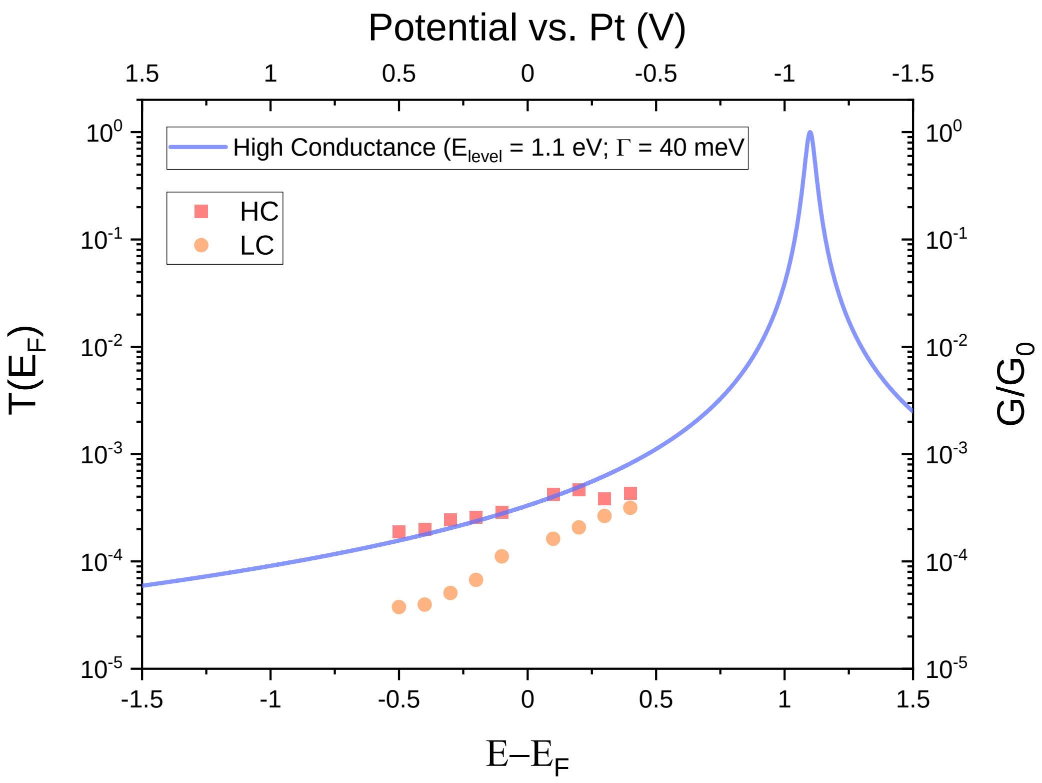

Figure S18. Experimental conductance and calculated *T(E)* for Au-VDP-Au junction as a function of the applied gating potential vs Pt quasi reference electrode.

3.4. The preparation of the Ag/AgCl reference electrode.

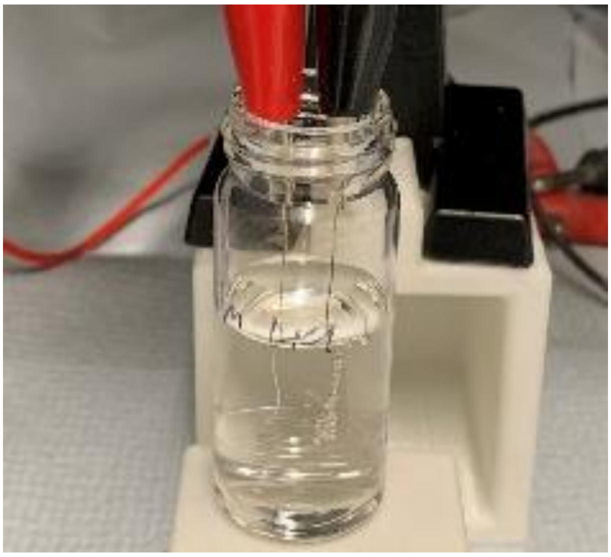

Figure S19. The homemade device for preparing the Ag/AgCl reference electrodes. Two Ag wires are held by the alligator clips. The red circuit wire is connected to the positive terminal of the 1.5 V AA battery and the black one to the negative end. An insoluble AgCl layer forms on the positive terminal, and H_2 bubbles can be seen on the negative side.

4. References

1. Xu, B.; Tao, N. J., Measurement of Single-Molecule Resistance by Repeated Formation of Molecular Junctions. *Science* **2003,** *301* (5637), 1221-1223.
2. Fink, C.; Laurenczy, G., CO_2 as a hydrogen vector – transition metal diamine catalysts for selective HCOOH dehydrogenation. *Dalton Trans.* **2017,** *46* (5), 1670-1676.
3. Gharib, B.; Hirsch, A., Synthesis and Characterization of New Ferrocene-Containing Ionic Liquids. *Eur. J. Org. Chem.* **2014,** *2014* (19), 4123-4136.
4. Kim, J.-H.; Kim, H. U.; Mi, D.; Jin, S.-H.; Shin, W. S.; Yoon, S. C.; Kang, I.-N.; Hwang, D.-H., Introduction of Perylene Units for Enhanced Interchain Interaction in Conjugated Polymers for Organic Photovoltaic Devices. *Macromolecules* **2012,** *45* (5), 2367-2376.
5. Li, W.; Zhao, Z.; Hu, W.; Cheng, Q.; Yang, L.; Hu, Z.; Liu, Y. A.; Wen, K.; Yang, H., Design of Thiazolo[5,4-d]thiazole-Bridged Ionic Covalent Organic Polymer for Highly Selective Oxygen Reduction to H2O2. *Chem. Mater.* **2020,** *32* (19), 8553-8560.
6. Dolomanov, O. V.; Bourhis, L. J.; Gildea, R. J.; Howard, J. A. K.; Puschmann, H., OLEX2: a complete structure solution, refinement and analysis program. *J. Appl. Crystallogr.* **2009,** *42*, 339-341.
7. Sheldrick, G., A short history of SHELX. *Acta Crystallogr., Sect. A* **2008,** *64* (1), 112-122.
8. Mészáros, G.; Li, C.; Pobelov, I.; Wandlowski, T., Current measurements in a wide dynamic range—applications in electrochemical nanotechnology. *Nanotechnology* **2007,** *18* (42), 424004.
9. Brooke, R. J.; Jin, C.; Szumski, D. S.; Nichols, R. J.; Mao, B.-W.; Thygesen, K. S.; Schwarzacher, W., Single-Molecule Electrochemical Transistor Utilizing a Nickel-Pyridyl Spinterface. *Nano Lett.* **2015,** *15* (1), 275-280.
